# Antimicrobial Activity of Ceftazidime-Avibactam, Ceftolozane-Tazobactam, Cefiderocol, and Novel Darobactin Analogs against Multidrug-Resistant Pseudomonas aeruginosa Isolates from Pediatric and Adolescent Cystic Fibrosis Patients

**DOI:** 10.1128/spectrum.04437-22

**Published:** 2023-01-24

**Authors:** Michael Marner, Laura Kolberg, Julia Horst, Nils Böhringer, Johannes Hübner, I Dewa M. Kresna, Yang Liu, Ute Mettal, Lei Wang, Melanie Meyer-Bühn, Sanja Mihajlovic, Matthias Kappler, Till F. Schäberle, Ulrich von Both

**Affiliations:** a Fraunhofer Institute for Molecular Biology and Applied Ecology (IME), Branch for Bioresources, Giessen, Germany; b Justus-Liebig-University of Giessen, Giessen, Germany; c Department of Pediatrics, Dr. von Hauner Children’s Hospital, University Hospital, LMU Munich, Munich, Germany; d Institute for Medical Information Processing, Biometry, and Epidemiology – IBE, LMU Munich, Munich, Germany; e Pettenkofer School of Public Health, Munich, Germany; f German Center for Infection Research (DZIF), Partner Site Giessen-Marburg-Langen, Giessen, Germany; g German Center for Infection Research (DZIF), Partner Site Munich, Munich, Germany; The University of North Carolina at Chapel Hill

**Keywords:** Gram-negative antibiotics, BamA, AMR, *Pseudomonas*, antibiotics, *Pseudomonas aeruginosa*, cystic fibrosis, natural antimicrobial products

## Abstract

The emergence and spread of antimicrobial resistance (AMR) in Gram-negative pathogens, such as carbapenem-resistant Pseudomonas aeruginosa, pose an increasing threat to health care. Patients with immunodeficiencies or chronic pulmonary disease, like cystic fibrosis (CF), are particularly vulnerable to Pseudomonas infections and depend heavily on antibiotic therapy. To broaden limited treatment options, this study evaluated the potency of the recently licensed drugs ceftazidime-avibactam (CZA), ceftolozane-tazobactam (C/T), and cefiderocol (FDC) as well as two novel preclinical antibiotics, darobactins B (DAR B) and B9 (DAR B9), against clinical P. aeruginosa isolates derived from respiratory samples of CF patients. We observed high levels of resistance to all three newly licensed drugs, with cefiderocol exhibiting the best activity. From the 66 investigated P. aeruginosa isolates, a total of 53% were resistant to CZA, 49% to C/T, and 30% to FDC. Strikingly, 52 of the evaluated isolates were obtained from CF patients prior to market introduction of the drugs. Thus, our results suggest that resistance to CZA, C/T, and FDC may be due to preexisting resistance mechanisms. On the other hand, our two novel preclinical compounds performed better than (CZA and C/T) or close to (FDC) the licensed drugs—most likely due to the novel mode of action. Thus, our results highlight the necessity of global consistency in the area of antibiotic stewardship to prevent AMR from further impairing the potency of antibiotics in clinical practice. Ultimately, this study demonstrates the urgency to support the development of novel antimicrobials, preferably with a new mode of action such as darobactins B and B9, two very promising antimicrobial compounds for the treatment of critically ill patients suffering from multidrug-resistant Gram-negative (MRGN) infections.

**IMPORTANCE** Antimicrobial resistance (AMR) represents an ever increasing threat to the health care system. Even recently licensed drugs are often not efficient for the treatment of infections caused by Gram-negative bacteria, like Pseudomonas aeruginosa, a causative agent of lung infections. To address this unmet medical need, innovative antibiotics, which possess a new mode of action, need to be developed. Here, the antibiogram of clinical isolates derived from cystic fibrosis patients was generated and new bicyclic heptapeptides, which inhibit the outer membrane protein BamA, exhibited strong activity, also against multidrug-resistant isolates.

## INTRODUCTION

Antibiotics save millions of lives every year and play a key part in medical care for critically ill or particularly vulnerable patients. However, irrational use of antibiotics (i.e., “bug-drug-mismatch,” “double-cover,” inadequate dose or route of administration, not performing therapeutic drug monitoring in cases where it is required, or unnecessarily prolonged antibiotic treatment) continues to hamper these beneficial effects. As a result, the emergence and global spread of antimicrobial resistance (AMR) impair the lifesaving potency of antimicrobial drugs including reserve antibiotics (1). AMR has been named as one of the top 10 threats to global health (2, 3). This antibiotic crisis poses unprecedented challenges to our health care system and is estimated to claim 10 million lives per year by 2050 (4–7). Pronounced use of antibiotics during the COVID-19 pandemic might even aggravate the situation (8). While bacteria evolve toward drug resistance, discovery and development of novel antibiotics against the WHO’s top-priority or “critical” Gram-negative pathogens (including carbapenem-resistant Pseudomonas aeruginosa) continue to challenge the scientific community.

Pseudomonas aeruginosa infections are particularly life-threatening for patients with severe lung diseases like cystic fibrosis (CF) (9). In CF patients, a genetically mediated disorder causes mucus to build up and damage organs, particularly the lungs and exocrine pancreas. The disease is caused by mutations in the cystic fibrosis transmembrane conductance regulator (CFTR) gene affecting the normal production or functioning of the CFTR protein found in the cells of the lungs and other parts of the body. Hence, this malfunctioning of mucus production and clearance can lead to permanent lung damage and favor respiratory infections by opportunistic pathogens such as P. aeruginosa (9).

Additive effects of neutrophilic hyperinflammation and virulence factors of Pseudomonas promote scar tissue (fibrosis) formation, respiratory failure, and ultimately death (10). Infected CF patients face a 2.6-times-higher risk of death over an 8-year period (11). Prevention, control and eradication of P. aeruginosa infections in CF patients rely on frequent and prolonged antibiotic therapy (intravenous as well as inhalational) in combination with intensified respiratory physiotherapy. As a consequence of frequent antibiotic use, multidrug-resistant (MDR) strains evolve and make the treatment of Pseudomonas infections in this vulnerable group of patients increasingly challenging.

Multidrug-resistant Gram-negative (MRGN) P. aeruginosa is resistant to the lead compounds of at least three (out of four) of the most relevant classes of Pseudomonas-active antibiotics in clinical practice (as defined by the German Commission for Hospital Hygiene and Infection Prevention [KRINKO]) (12). If a P. aeruginosa strain is classified as MRGN, antimicrobial treatment is limited to reserve antibiotics (such as colistin) combined with traditional antipseudomonal agents. Unfortunately, combinatorial therapy is accompanied by an increased risk of antibiotic toxicity and secondary infections, particularly in view of prolonged treatment courses in CF patients (13, 14). In this context, new developments in antibiotic treatment of MRGN P. aeruginosa are urgently needed.

Drugs recently introduced into clinical practice comprise structural modifications of well-known antibiotic classes (15, 16), combinatorial drugs composed of a β-lactamase inhibitor and a cephalosporin (17) (or carbapenem) (18), and the catechol-cephalosporin cefiderocol (19, 20). The number of innovative anti-Gram-negative compounds with a new molecular target or mode of action, and hence fewer (if any) prevalent resistance mechanisms, is even smaller. One promising antibiotic with an unique mode of action is the recently discovered darobactin A (DAR A) (21). The antibiotic inhibits the functionality of the multiprotein β-barrel-assembly-machinery complex (Bam) by binding to the transmembrane protein BamA. DAR A exhibits excellent *in vitro* and *in vivo* activity against Gram-negative pathogens such as Escherichia coli and Klebsiella pneumoniae (21). Unfortunately, DAR A is less potent against P. aeruginosa strains, the only exception being the moderately virulent laboratory strain PAO1 (22) (MIC, 2 to 4 μg/mL). Mutasynthetic derivatization and biosynthetic pathway engineering expanded the group of darobactins and unearthed two analogs (DAR B [23] and DAR 9 [24]) with enhanced potency, also against P. aeruginosa. The two serine residues within the core peptide sequence of DAR A are replaced by threonine at position four and arginine at position six in DAR B. In DAR 9 the phenylalanine at position seven is replaced by tryptophan, while the remaining core peptide is identical to that in DAR A.

In this study, we evaluated the *in vitro* activity of currently available reserve antibiotics (ceftazidime-avibactam [CZA], ceftolozane-tazobactam [C/T], and cefiderocol [FDC]) against MRGN P. aeruginosa strains from a pediatric and adolescent cystic fibrosis cohort. The results allowed a thorough characterization according to the latest clinical breakpoints published by EUCAST. Furthermore, we describe the bioactivity-driven design and production of darobactin B9 (DAR B9), which combines the amino acids W^1^N^2^W^3^T^4^K^5^R^6^ of DAR B and W^7^ of DAR 9, followed by focused profiling against the clinical isolates. The antimicrobial potency of DAR B9 was compared to the activity of DAR B, CZA, C/T, and FDC.

## RESULTS

### Collection and characterization of the P. aeruginosa library.

Between 2006 and 2018 a total of 66 P. aeruginosa strains were isolated from CF patients at the Dr. von Hauner Children’s Hospital (Munich, Germany). Strains were classified by plating on selective agar medium according to the gold standard method recommended by the European Pharmacopoeia. Briefly, cetrimide agar was used for genus determination (Pseudomonas spp.) and blood agar for differentiation between Pseudomonas fluorescens and P. aeruginosa. The number of isolates from each of the 35 patients varied from 1 to 8 isolates (Table 1). The majority (57%) contributed one isolate. The study population was 60% female and 46% homozygous for the delta F508 mutation at the NBD 1 subdomain of CFTR. Age at initial P. aeruginosa infection ranged from 3 months to 22 years, with a median age of 8 years. A detailed account of demographic and clinical data collected for each patient is given in Table S1 in the supplemental material.

**TABLE 1:** Distribution of demographic and clinical data for 35 patients

Type of data	No. (%) of patients
Sex	
Male	14 (40.0)
Female	21 (60.0)
Age in yr at first Pseudomonas aeruginosa colonization^*a*^	
0–2	5 (14.3)
3–6	6 (17.1)
7–11	9 (25.7)
12–17	8 (22.9)
18–22	2 (5.7)
No. of isolates collected	
1	20 (57.0)
2	8 (2.9)
3	1 (2.9)
4	4 (11.4)
7	1 (2.9)
8	1 (2.9)
Genetics: delta F508 mutation^*b*^	
Homozygote	16 (45.7)
Heterozygote	9 (25.7)
Other mutations	7 (20.0)

a Unknown information in 5 (14.3%) patients.

b Unknown information in 3 (8.6%) patients.

Microbiological quality controls (QCs) of the P. aeruginosa isolates obtained from the CF patients included phylogenetic (16S rRNA gene sequencing) and biochemical (Vitek 2 automated system equipped with a GN ID card) identification. In addition to the data obtained at the time of isolation, new resistance profiles were determined and used to reclassify the isolates according to the latest clinical breakpoints published by EUCAST (v 12.0). In detail, we used piperacillin, ceftazidime, cefepime, ciprofloxacin, meropenem, imipenem, tobramycin, and colistin for resistance profiling of each strain (Table S2). A set of 38 isolates (58%) were reclassified as 4-MRGN strains, thus being resistant to lead compounds of all four antibiotic classes tested. Another 10 isolates (15%) were found to be 3-MRGN. Within the 3-MRGN group, 4 strains were “susceptible with increased exposure” (I) to ciprofloxacin and 6 to carbapenems (either imipenem or meropenem). No 3-MRGN strain was susceptible at standard dosage (S) to any of the tested drugs. The remaining 18 (27%) isolates were classified as non-MRGN. Of 49 isolates for which susceptibility testing against tobramycin was performed, 19 (39%) were resistant. None of the 49 tested strains exhibited resistance to colistin (Table S2).

### Susceptibility testing of recently licensed substances CZA, C/T, and FDC.

**FIG 1:**
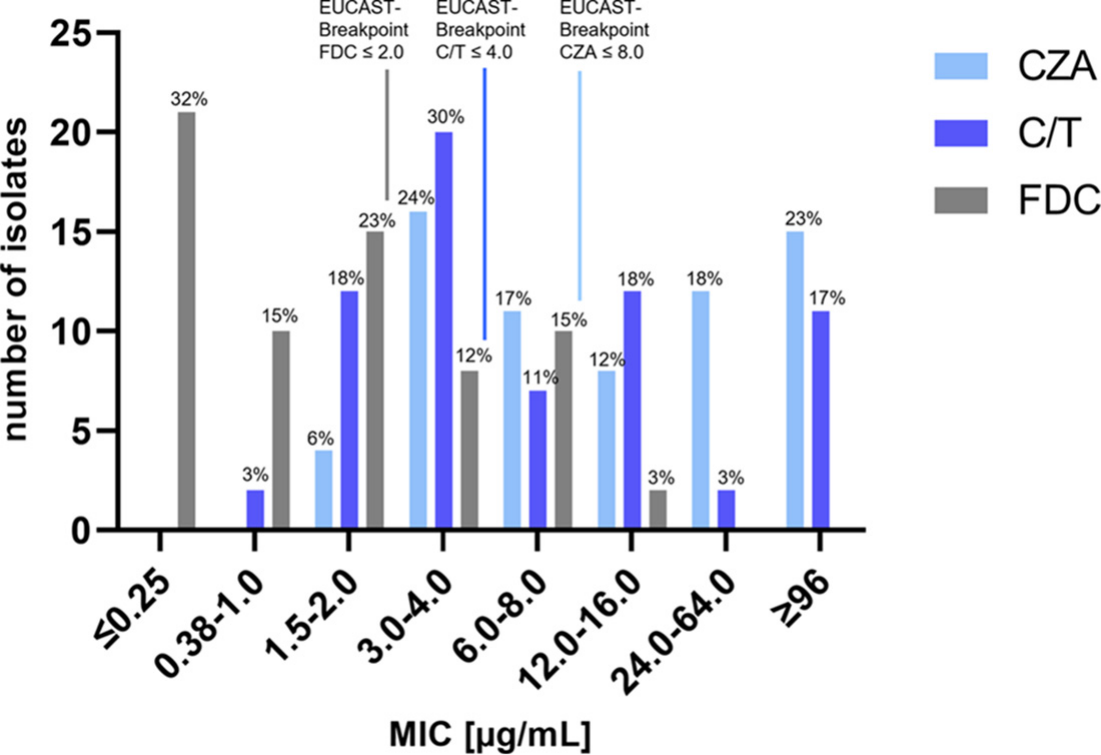
MIC distribution of ceftazidime-avibactam (CZA), ceftolozane-tazobactam (C/T), and cefiderocol (FDC) for 66 Pseudomonas aeruginosa isolates. Breakpoints determined by the European Committee on Antimicrobial Susceptibility Testing (EUCAST) were used to define bacterial isolates as resistant (R) or susceptible (S).

In addition to the susceptibility tests used for MRGN classification of all 66 isolates, the MIC distribution of recently licensed CZA, C/T, and FDC was determined by Etest and interpreted using the latest EUCAST breakpoints (Fig. 1; Table S3). Overall, a significantly lower share of isolates was resistant to FDC than to the other two drugs (30% FDC, 53% CZA, and 49% C/T; *P* value, <0.01). Interestingly, the complete set of analyzed P. aeruginosa strains was isolated from CF patients (outpatients and inpatients) prior to drug marketing authorization of FDC and C/T and hence introduction to medical care facilities in Germany (25, 26). Nonetheless, a total of 20 isolates was resistant to FDC and almost half of the strains were resistant to C/T (*n* = 32). For CZA, 52 out of the 66 strains were sampled before market introduction in 2016 (27). However, all resistant isolates (*n* = 35) were sampled before May 2015.

**FIG 2:**
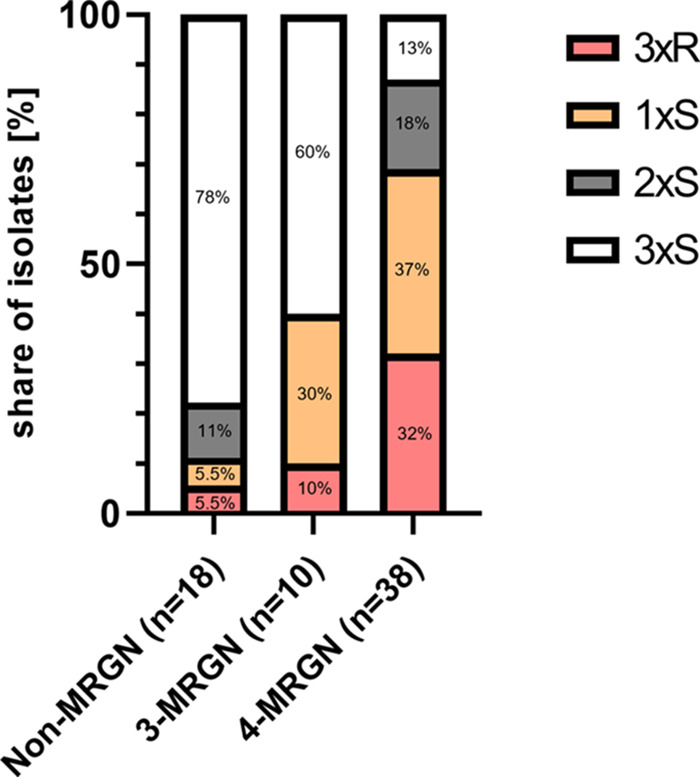
Distribution of susceptibility patterns to ceftazidime-avibactam (CZA), ceftolozane-tazobactam (C/T), and cefiderocol (FDC) of 66 Pseudomonas aeruginosa isolates by type of MRGN. 3xR/3xS, share of isolates that were resistant (R)/susceptible (S) to all three antibiotics tested (CZA, C/T, and FDC); 2xS and 1xS, proportion of isolates with susceptibility to two or one of the antibiotics tested, respectively.

Antimicrobial susceptibility results for all isolates, stratified by MRGN type, are listed in Table 2. Overall, isolates classified as 3-/4-MRGN showed lower susceptibility toward CZA, C/T, and FDC (Fig. 2; Table S2). FDC showed the highest overall *in vitro* activity (MIC_50_ = 1 μg/mL and MIC_90_ = 6 μg/mL) compared to CZA (MIC_50_ = 8 μg/mL, MIC_90_ = >256 μg/mL), and C/T (MIC_50_ = 4 μg/mL, MIC_90_ = 96 μg/mL). Isolates classified as non-MRGN had similar susceptibility rates for CZA (89%), C/T (83%), and FDC (89%), but the MIC_50_ and MIC_90_ of CZA (4 and 16 μg/mL) were higher than those for C/T (MIC_50_ = 3 μg/mL, MIC_90_ = 12 μg/mL) and FDC (MIC_50_ = 0.25 μg/mL, MIC_90_ = 3 μg/mL). In 3-MRGN isolates, FDC had the highest proportion of susceptible isolates (80% FDC, 70% C/T, and 60% CZA) as well as the lowest MIC_50_ (0.25 μg/mL FDC, 4 μg/mL CZA and C/T) and MIC_90_ (6 μg/mL FDC, 24 μg/mL C/T, and 64 μg/mL CZA). Among 4-MRGN isolates, FDC had a higher susceptibility rate (58%) than C/T (32%) and a significantly higher susceptibility rate than CZA (24%) (Bonferroni-corrected *P* value, <0.02). The MIC_50_ (2 μg/mL) and MIC_90_ (8 μg/mL) were also lower for FDC than for CZA (MIC_50_ = 32 μg/mL, MIC_90_ = >256 μg/mL) and C/T (MIC_50_ = 12 μg/mL MIC_90_ = >256 μg/mL) in 4-MRGN isolates. For FDC, the susceptibility differences between MRGN types were not significant. In contrast to that, susceptibility decreased with higher MRGN types for CZA and C/T. In particular, comparison of susceptibility between non-MRGN (89% CZA, 83% C/T) and 4-MRGN (24% CZA, 32% C/T) isolates showed a significant difference (Bonferroni-corrected *P* value for both compounds, <0.01).

**TABLE 2:** Results of antimicrobial susceptibility testing with ceftazidime-avibactam (CZA), ceftolozane-tazobactam (C/T), and cefiderocol (FDC) for 66 Pseudomonas aeruginosa isolates stratified by MRGN^*a*^ classification

Total or MRGN^*a*^ type (*n*)	MIC_50_^*b*^			MIC_90_^*b*^			No. (%) of isolates susceptible^*c*^		
	CZA	C/T	FDC	CZA	C/T	FDC	CZA	C/T	FDC
Total (66)	8	4	1	>256	96	6	31 (47)	34 (52)	46 (70)
Non-MRGN (18)	4	3	0.25	16	12	3	16 (89)	15 (83)	16 (89)
3-MRGN (10)	4	4	0.25	64	24	6	6 (60)	7 (70)	8 (80)
4-MRGN (38)	32	12	2	>256	>256	8	9 (24)	12 (32)	22 (58)

a MRGN (multidrug-resistant Gram-negative) bacteria are classified according to KRINKO (the German Commission for Hospital Hygiene and Infection Prevention) as unsusceptible to the lead compounds of 3 (3-MRGN) or 4 (4-MRGN) of the following antibiotic classes: acyl ureidopenicillins (piperacillin-tazobactam or piperacillin), third- or fourth-generation cephalosporins (ceftazidime and cefepime), fluoroquinolones (ciprofloxacin), and carbapenems (meropenem and imipenem) (12).

b MIC_50/90_ is defined as the MIC in micrograms per milliliter at which 50%/90% of isolates were inhibited, respectively.

c Susceptibility breakpoints determined by the European Committee on Antimicrobial Susceptibility Testing (EUCAST) were 8 μg/mL for CZA, 4 μg/mL for C/T, and 2 μg/mL for FDC, respectively.

Distribution of susceptibility patterns (to CZA, C/T, and FDC) was compared across MRGN classes (Fig. 2; Table S4). Of all non-MRGN isolates, the largest proportion was susceptible to all three antibiotics (78%). This group (3xS) declined with higher MRGN types to 60% for 3-MRGN and 13% for 4-MRGN. On the other hand, the fraction of isolates with susceptibility to only one (1xS) or two (2xS) antibiotics tested (30% for 3-MRGN and 55% for 4-MRGN) was higher in MRGN isolates than in non-MRGN isolates (16.5%). Resistance against all three antibiotics was observed in 32% of 4-MRGN isolates. The antibiotic susceptibility of 4-MRGN isolates was observed to be significantly lower than that of non-MRGN isolates (Bonferroni-adjusted *P* value, <0.001).

### Design and production of darobactin analogs.

**FIG 3:**
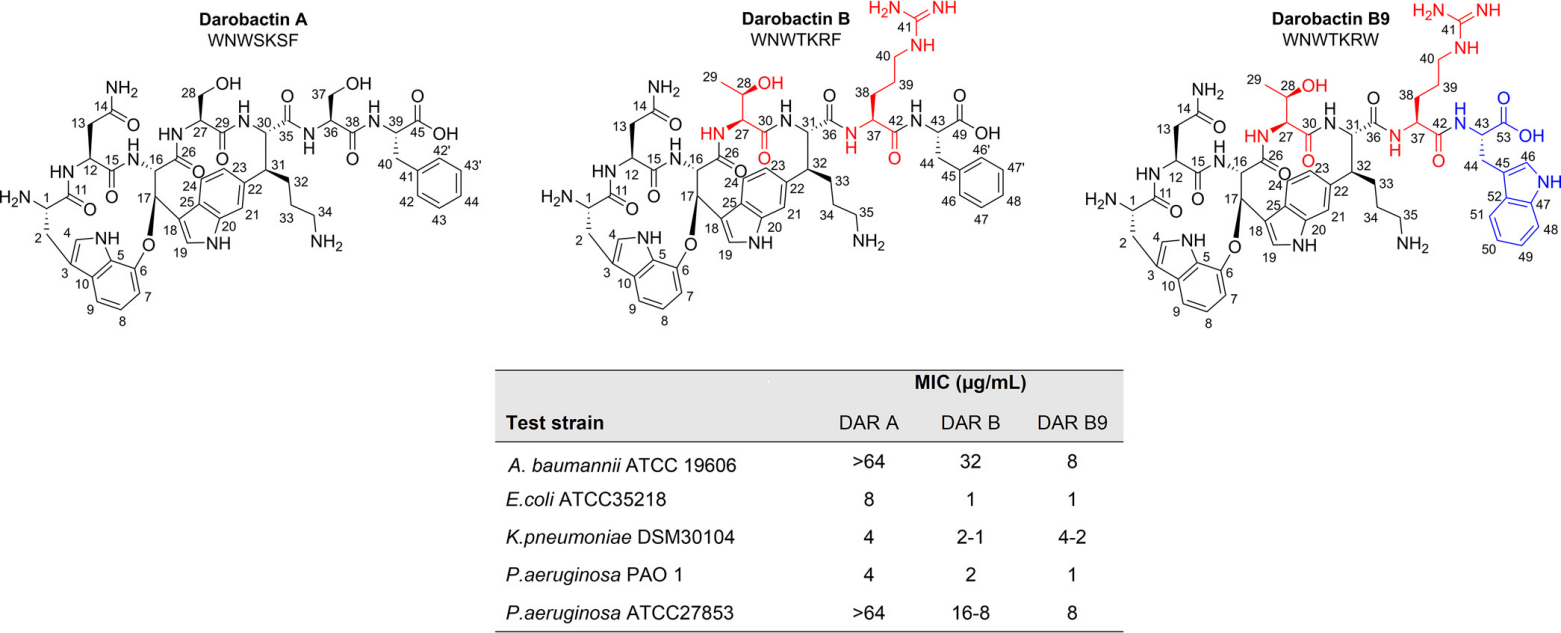
Structures of DAR A, DAR B, and newly designed DAR B9 and bioactivity against Gram-negative indicator strains. Distinguishing features of darobactin B are serine replacements by threonine and arginine at positions 4 and 6, respectively. In darobactin B9, phenylalanine is additionally replaced by tryptophan at the C-terminal position of the heptapeptide. DAR B and DAR B9 exhibit pronounced activity against the test panel. Data for DAR A and DAR B were obtained from previous studies.

The bicyclic heptapeptide DAR A exhibits compelling efficacy against Gram-negative pathogens. However, in contrast to E. coli and K. pneumoniae, many P. aeruginosa isolates show elevated MICs (21). Hence, we aimed to design optimized analogs. We heterologously expressed and tested darobactin variants identified in genome sequences of *Photorhabdus* sp. and *Yersinia* sp. and found DAR B to be more active (23). However, the number of darobactin-like biosynthetic gene clusters in published genomes is limited. To get access to further (not naturally occurring) darobactin analogs, we decided to engineer DAR B variants by codon exchange on the expression vector. Darobactin blocks outer membrane protein (OMP) maturation by binding at the same position on the BamA strand 1β as the signal sequence of the native substrates. As most β-signal peptide sequences of OMPs exhibit a conserved C-terminal aromatic amino acid residue, which was shown to be essential for recognition and processing of the OMP by BamA (28, 29), we primarily focused on exchanging F^7^ with W^7^ within in the core peptide sequence of DAR B. In fact, an F^7^/W^7^ exchange at the C terminus of DAR A was independently reported to possess better activity than the natural DAR A (24). Ultimately, we were able to produce DAR B9 (W^1^N^2^W^3^T^4^K^5^R^6^W^7^), which exhibits three amino acid substitutions compared to DAR A (Fig. 3).

The compounds were produced using our heterologous expression system described previously (30). The structure of the new derivative DAR B9 was validated by thorough tandem mass spectrometry (MS/MS), as well as 1- and 2-dimensional nuclear magnetic resonance (NMR) spectroscopy experiments (for fragmentation pattern annotation and key correlations, see Fig. S1 to S22 and Table S5). Briefly, NMR data comparison between DAR B and B9 illustrates the expected disappearance of the phenylalanine signals, while at the same time new signals originating from the tryptophan residue are observed (Fig. S3 and S4). In the ^1^H-NMR new signals were observed at 7.72 (H-51), 7.57 (H-48), 7.31 (H-46), 7.29 (H-49), and 7.21 (H-50) ppm, corresponding to the aromatic protons of the indolyl system. Furthermore, the signals for the protons in α- and β-positions (H-43 and H-44) were slightly shifted (DAR B9, 4.69 [H-43], 3.41 and 3.314 [H-44] ppm; DAR B, 4.77 [H-43], 3.33 and 3.17 [H-44] ppm). The most remarkable features in the ^13^C-NMR spectrum were the considerably shifted signals observed for the carbonyl group (DAR B9, 180.5 ppm; DAR B, 179.0 ppm), the methylene group (DAR B9, 31.3 ppm; DAR B, 41.0 ppm), and the quaternary carbon in γ-position (DAR B9, 113.9 ppm; DAR B, 141.1 ppm) of the tryptophan residue, along with the appearance of new signals at 140.7 (C-47), 131.7 (C-52), 129.2 (C-46), 126.5 (C-49), 123.9 (C-50), 123.1 (C-51), and 116.4 (C-48) ppm. At the same time, the signals of the phenyl residue (C-46/46′ [133.9 ppm], C-47/47′ [133.2 ppm], and C-48 [131.7 ppm]) were not visible anymore.

Primary activity profiling against a panel of Gram-negative indicator strains showed enhanced activity of DAR B9 compared to our previous observation for DAR B and DAR A (21, 23). Most strikingly, our results indicate that the potency of DAR B9 against Acinetobacter baumannii and P. aeruginosa is at least 8-fold higher than that of DAR A. Intrigued by these results, we profiled DAR B and B9 against the clinical CF P. aeruginosa isolates.

### Activity profiling of darobactins B and B9 against P. aeruginosa isolates.

**FIG 4:**
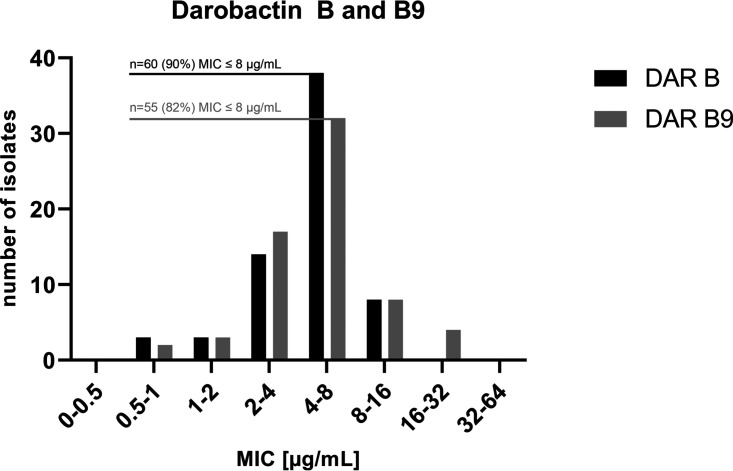
MIC distribution of DAR B and DAR B9 for 66 clinical Pseudomonas aeruginosa isolates plus the EUCAST quality control strain ATCC 27853.

Overall, both compounds, darobactin B and darobactin B9, showed promising antimicrobial activity against our clinical P. aeruginosa test panel (Fig. 4). No statistically significant difference between DAR B and B9 was observed (*P* value = 0.32).

The majority of isolates could not tolerate more than 8 μg/mL of either compound (90% DAR B and 82% DAR B9). Hence, we observed identical total MIC_50_ values (8 μg/mL) and only slightly different total MIC_90_ values (8 and 16 μg/mL) for the two different darobactins (Table 3). These values are in accordance with our previous findings against the EUCAST quality control strain P. aeruginosa ATCC 27853 (also included in Fig. 3). The MIC_90_ values of DAR B showed a minor difference (1 dilution step) between the 4-MRGN (16 μg/mL) and the 3-MRGN as well as non-MRGN subgroups (both 8 μg/mL). DAR B9 exhibited a slightly higher MIC_90_ for the 3-MRGN subgroup (16 μg/mL), while the other values were identical compared to DAR B (Table 3).

**TABLE 3:** Results of antimicrobial susceptibility testing with darobactin B and darobactin B9 for 66 P. aeruginosa isolates stratified by MRGN^*a*^ classification

Total or MRGN^*a*^ type (*n*)	MIC_50_^*b*^		MIC_90_^*b*^	
	DAR B	DAR B9	DAR B	DAR B9
Total (66)	8	8	8	16
Non-MRGN (18)	8	8	8	8
3-MRGN (10)	8	8	8	16
4-MRGN (38)	8	8	16	16

a MRGN (multidrug-resistant Gram-negative) bacteria are classified according to KRINKO as unsusceptible to the lead compounds of 3 (3-MRGN) or 4 (4-MRGN) of the following antibiotic classes: acyl ureidopenicillins (piperacillin-tazobactam or piperacillin), third- or fourth-generation cephalosporins (ceftazidime and cefepime), fluoroquinolones (ciprofloxacin), and carbapenems (meropenem and imipenem).

b MIC_50/90_ is defined as the MIC in micrograms per milliliter at which 50%/90% of isolates were inhibited, respectively (12).

Remarkably, the total MIC_90_ of both darobactins is substantially lower than those of the licensed drugs CZA (>256 μg/mL) and C/T (96 μg/mL). FDC was slightly more efficacious, but the difference from DAR B was observed to be within the range of 1 dilution step across all MRGN classes.

## DISCUSSION

Reserve antibiotics are of key importance to any clinician treating patients with multidrug-resistant Pseudomonas infections. Cystic fibrosis patients are at particular risk of developing severe exacerbations due to respiratory tract infections—significantly reducing quality of life and survival rates. To support the development of a broader range of treatment options, this study aimed to evaluate the potency of (i) the recently licensed antibiotic agents CZA, C/T, and FDC as well as (ii) two preclinical compounds, which belong to the recently discovered family of darobactins, against a set of P. aeruginosa isolates from a pediatric and adolescent cystic fibrosis cohort.

We observed high levels of resistance to all three newly licensed drugs in our non-MRGN and MRGN P. aeruginosa collection. Although FDC still exhibited good activity across all subgroups, this study shows that almost a third of the 4-MRGN isolates were resistant to all three drugs tested. Remarkably, 52 test strains exhibited resistance although being isolated prior to market introduction of the tested drugs. Thus, our results suggest that resistance to CZA, C/T, and—to a lesser extent—FDC may be due to preexisting resistance mechanisms.

This phenomenon might in part be due to the molecular target of these drugs. All three drugs (CZA, C/T, and FDC) ultimately inhibit the transpeptidase activity of penicillin-binding proteins (PBPs) to arrest the peptidoglycan biosynthesis. In Pseudomonas, common mechanisms to protect the PBP enzyme comprise the biosynthesis of β-lactamases, efflux pumps, and target modulation as well as prohibition of drug entrance into the periplasmic space. CZA and C/T are specifically developed to combat β-lactamase-induced resistance to β-lactams. The β-lactamase-inhibiting agents avibactam and tazobactam enable PBP inhibition by the respective cephalosporin component of the drugs. Unfortunately, no approved β-lactamase inhibitor, including avibactam and tazobactam, exhibits activity toward metallo-β-lactamases (MBLs) and class D carbapenemases, rendering the cephalosporin unprotected from hydrolysis (31, 32). In addition, mutations in broadly distributed β-lactamase genes such as *bla*_SHV_ and *bla*_CTX−M_ have been reported to contribute to CZA resistance (33, 34). Cross-resistance between CZA and C/T is known in the literature (31). In that sense it is not surprising that the efficacy of CZA and C/T was particularly poor in the 4-MRGN subgroup (MIC_90_, >256 μg/mL).

FDC had significantly higher overall susceptibility rates across all MRGN subgroups. It is known that FDC exhibits improved stability against β-lactamase (including MBL)-induced hydrolysis and is able to circumvent outer membrane-mediated exclusion. By mimicking catecholate-type siderophores, the compound can enter the periplasm via iron transporter channels where it binds to PBP (32). Although FDC exhibited intriguing activity, e.g., its MIC_90_ is 6 μg/mL, the high resistance rate (42%) in 4-MRGN strains is alarming. The traditional molecular target and structural similarity to long-established drugs might facilitate resistance development toward FDC. In fact, a number of reports indicate that FDC resistance is increasing, an effect that may in part be due to irrational use of this important reserve drug (35, 36). Hence, clinicians need to be aware that prior to using any newly licensed substance, susceptibility testing of the respective clinical isolates is of key importance to attain the desired antimicrobial efficacy.

The high resistance rates demonstrated for CF P. aeruginosa isolates against reserve antibiotics clearly illustrate the critical need for further treatment options. Considering this, we profiled a recently discovered natural product class that has the potential to be developed into a new antibiotic.

The novel compounds darobactin B and darobactin B9 demonstrated promising antimicrobial activity across all MRGN subgroups. To date, the compound family has been in preclinical development but has nevertheless performed already better than (CZA and C/T) or similarly to (FDC) recently licensed drugs to treat P. aeruginosa infections in CF patients. It can be expected that further optimization of these frontrunners will result in increased potency. Furthermore, we observed that the potency of DAR B and B9 was not impaired by prevalent resistance mechanisms—most likely due to the suggested novel mode of action and molecular target of the compounds. In addition, it has been shown before that the frequency of resistance of E. coli to DAR A is rather low (8 × 10^−9^) and, most interestingly, that the resistant strains lost their virulence in mouse infection models (21). Further studies on the precise mode of action and resistance development should be carried out for DAR B9 and compared to the data for DAR A and B.

Thus, speeding up the process for clinical trials and eventual licensing of these novel antibiotic compounds should be a priority, since clinicians worldwide will soon be left without any choice for treating multidrug-resistant Pseudomonas infections. That is particularly true for CF patients, as initial MRGN colonization frequently appears at a young age. However, only CZA and C/T, both showing lower potency in our study, are currently approved by the European Medicines Agency for use in children. Further treatment options for young CF patients colonized with MRGN P. aeruginosa remain an unmet medical need.

Our study is one of many calls to action in the area of (i) antimicrobial stewardship and (ii) development of novel antibiotic compounds. Antibiotics need to be handled with care, meaning that rational use of antibiotics has to become a mandatory principle in hospital and community settings alike. Furthermore, new drugs effective against MDR pathogens are urgently needed. Development and market introduction must be actively supported by political and medical bodies. In particular, antibiotics with a novel mode of action, such as the frontrunner molecules tested in this study, are of highest interest. Funding schemes must be put in place to accelerate development of such lifesaving drugs. Only if these approaches go hand in hand and are followed coherently across all countries may we preserve the potency of antimicrobial drugs for the health care systems of the future.

## MATERIALS AND METHODS

### Bacterial isolates.

This retrospective, monocentric analysis was conducted using MRGN P. aeruginosa isolates obtained from routinely collected respiratory samples from CF patients at the Dr. von Hauner Children’s Hospital in Munich, Germany. As part of the LMU University Hospital, the Department of Pediatric Pneumology is one of the oldest and largest Cystic Fibrosis Centers in Germany. For more than 40 years, around 350 children and adults diagnosed with CF have received regular outpatient and inpatient care here every year (37). During these routine appointments, respiratory material (sputum, throat swab, or bronchoalveolar lavage [BAL] fluid) was collected from patients and analyzed within microbiological examinations. The phenotypic classification of isolates was done by plating on selective medium according to the gold standard method recommended by the European Pharmacopoeia. The phenotypic classification of isolates was done by plating on selective medium according to the gold standard method recommended by the European Pharmacopoeia. First, samples were incubated on Pseudomonas-selective agar (cetrimide agar). Then, the bacteria were cultured on Columbia blood agar for 1 day at 42°C to differentiate between P. aeruginosa (growth) and P. fluorescens (no growth). Between 2006 and 2018, when an MRGN P. aeruginosa strain was detected and identified as clinically relevant, isolates were stored at −20°C in cryovials (Mast Group, Reinfeld, Germany). For this study, 66 P. aeruginosa samples collected during this period were examined.

The bacterial samples were reactivated on sheep’s blood agar plates (BD Diagnostics, Heidelberg, Germany). Quality control was done by 16S rRNA sequencing (GenBank accession numbers OP737540 to OP737605; see Table S2 in the supplemental material) and a Vitek 2 automated system equipped with a GN ID card (bioMérieux, Marcy-l’Etoile, France), respectively. For all P. aeruginosa isolates, new resistance profiles were measured with Vitek 2 and interpreted according to the clinical breakpoints determined by the European Committee on Antimicrobial Susceptibility Testing (EUCAST) (v 12.0) (38). The resistance profiles were used to reclassify the isolates into MRGN categories according to classification guidelines published by the German Commission for Hospital Hygiene and Infection Prevention (KRINKO) at the Robert Koch Institute. Briefly, the MRGN categories define multidrug-resistant Gram-negative (MRGN) P. aeruginosa as resistant to the lead compounds of 3 (3-MRGN) or 4 (4-MRGN) of the following antibiotic classes: acyl ureidopenicillins (piperacillin or piperacillin-tazobactam), third- or fourth-generation cephalosporins (ceftazidime and cefepime), fluoroquinolones (ciprofloxacin), and carbapenems (meropenem and imipenem) (12).

### Ethics.

This study was approved by the Ethics Committee of the Ludwig Maximilians University Munich under project number 21-0334.

### Demographic and clinical data acquisition.

Demographic and clinical data of patients from whom P. aeruginosa isolates had been detected in respiratory samples were obtained from electronic and paper-based medical records in the hospital database. Collected variables were sex, age at initial P. aeruginosa colonization, and presence of homozygosity or heterozygosity for the delta F508 (df508) mutation in the cystic fibrosis transmembrane regulator (CFTR) gene. For some patients, it was also possible to determine the date of initial MRGN P. aeruginosa colonization. In addition, for most isolates susceptibility to colistin and tobramycin was tested during antimicrobial susceptibility testing as part of QC and was thus also included as a variable in the analysis.

### Antimicrobial susceptibility testing.

The antimicrobial susceptibility of the CF isolates to CZA, C/T, and FDC was determined by gradient diffusion testing using Etest strips (Liofilchem, Roseto degli Abruzzi, Italy) according to the hospital’s laboratory standards. After subculturing the isolates overnight on blood agar plates (BD Diagnostics), isolated colonies were suspended in 0.45% saline and adjusted to McFarland turbidity standards (0.5 to 1.0). The dilutions were streaked onto the surface of Mueller-Hinton agar plates (BD Diagnostics) before Etest strips were applied. The inoculated agar plates were incubated for 16 to 24 h at 35 ± 2°C (39–41). MICs (micrograms per milliliter) were determined according to the manufacturer’s instructions (38). To ensure objectivity, MIC was read twice by two independent investigators and then checked for agreement. Finally, isolates were categorized as resistant or susceptible according to the latest EUCAST breakpoints (8 μg/mL for CZA, 4 μg/mL for C/T, and 2 μg/mL for FDC). Antimicrobial activity (of CZA, C/T, and FDC) was assessed as the MIC that still showed efficacy in 50% (MIC_50_) or 90% (MIC_90_) of the isolates tested and as the percentage of isolates classified as susceptible according to EUCAST breakpoints (susceptibility rate).

Antipseudomonal potency of darobactins B and B9 was assessed by broth microdilution assays with only minor modifications to previous reports (42–44). All compounds were dissolved in water and tested in triplicate. In the case of MIC variation within a triplicate, the higher MIC was used for analysis. For all tested strains the differences within MIC replicates were not higher than 1 dilution step. All strains were cultivated overnight (37°C, 180 rpm) in cation-adjusted Mueller-Hinton II medium (BD). The cell density of the overnight culture was adjusted to 5 × 10^5^ cells/mL. A dilution series of the standard antibiotics cefotaxime and gentamicin (64 to 0.03 μg/mL) was added to each assay plate. Cell suspensions without darobactin or antibiotic standards were used as growth controls. After assay incubation (18 h, 37°C, 180 rpm, 85% relative humidity [rH]), cell growth was determined by measuring the turbidity of each test well with a microplate spectrophotometer at 600 nm (LUMIstar Omega; BMG Labtech). The MIC was defined as the lowest concentration which inhibited the growth of the test strain by at least 80% relative to the growth control.

### Statistical analysis.

Distribution of collected variables was described in absolute numbers (*n*) and percentages (%) for categorical variables. For continuous variables, median and range were also calculated. Antimicrobial activity (of CZA, C/T, and FDC) was assessed as the MIC that still showed efficacy in 50% (MIC_50_) or 90% (MIC_90_) of the isolates tested and as the percentage of isolates classified as susceptible according to EUCAST breakpoints (susceptibility rate). The comparison of susceptibility rates for CZA, C/T, and FDC was performed by χ^2^ test or Fisher’s exact test. *P* values of less than 0.05 were considered significant. For pairwise comparisons, Bonferroni-adjusted *P* values were reported. All statistics were performed using the software R version 4.0.5. For darobactins B and B9 the contingency table contained the absolute number of isolates with MICs of ≤8 μg/mL and >8 μg/mL and the comparison was achieved by χ^2^ test and Fisher’s exact test in GraphPad Prism. *P* values of less than 0.05 were considered significant.

### Creation of pRSF-ADC5_WNWTKRW.

For the production of darobactin B9, the core region for the darobactin precursor peptide of pZW-ADC5 (30) was modified to encode WNWTKRW by amplification of the full pZW-ADC5 using the primers 5′-TGGTAAAGCTTATCCCATCAGGTTATTTTATTTTCCT-3′/5′-TGATGGGATAAGCTTTACCAACGTTTGGTCCAGTTCCAGGCCGTGATCTC-3′ and New England Biolabs (NEB) Q5 polymerase according to the manual. The PCR product was purified on a 1% Tris-agarose-EDTA (TAE) agarose gel using standard methodology and recircularized using homemade isothermal assembly master mix (45). E. coli TOP10 cells were transformed with the isothermal assembly mix by standard electroporation procedure and subsequently selected on LB_Kan_ (lysogeny broth supplemented with kanamycin) at 37°C overnight. Successful codon exchange was corroborated by sequencing, showing successful creation of the plasmid pRSF-ADC5_WNWTKRW. Subsequently, pRSF-ADC5_WNWTKRW was transferred to E. coli Rosetta as an expression strain by electroporation, and transformed cells were selected on LB_Kan/CM_ (lysogeny broth supplemented with kanamycin and chloramphenicol) at 37°C. Clones carrying the plasmid were grown in 5 mL LB_Kan/CM_ at 200 rpm overnight, and the culture was mixed 1:1 with 100% glycerol, aliquoted, and stored at −80°C until further use.

### Production of darobactin B9.

The production of darobactin B9 follows the production of darobactin B as previously described (23) with only minor modifications. Codon exchange on the expression vector was done to yield the product with the desired core peptide sequence W^1^N^2^W^3^T^4^K^5^R^6^W^7^. In brief, 2-L shaking flasks containing 1 L LB_Kan/CM_ were inoculated with 5% (vol/vol) E. coli Rosetta plus pRSF-ADC5_WNWTKRW overnight preculture (LB_Kan/CM_, 37°C, 200 rpm) and grown at 30°C, 200 rpm, to an optical density at 600 nm (OD_600_) of 0.5. Production of darobactin B9 was induced by the addition of isopropyl-β-d-thiogalactopyranoside (IPTG) to 1 mM, and fermentation continued for 6 days. Subsequently, cells and medium were separated by centrifugation and darobactin B9 was extracted from the supernatant by solid-phase extraction using 2% Amberlite XAD16N and eluted using 80% methanol (MeOH) and 100% MeOH. Methanol was evaporated to obtain the aqueous crude extract, which was loaded to a SP Sepharose XL (GE Healthcare) strong cation exchange column with a 220-mL bed volume. The column was washed with 10 column volumes (CV) H_2_O plus 0.1% formic acid (FA), eluted with 10 CV of 50 mM ammonium acetate buffer with pH 5, pH 7, pH 9, and pH 11, respectively, and washed with 10 CV 1 M NaCl and 1 M NaOH. All fractions were analyzed by liquid chromatography-mass spectrometry (LC-MS) using the setup as previously described (23), and darobactin B9-containing fractions were combined and dried. Final purification was performed by high-performance liquid chromatography (HPLC) (Agilent C_18_ 5 μm; 250 by 10 mm; Restek) using a suited gradient of MeCN/H_2_O plus 0.1% FA. Darobactin B9 was dried and stored for further use at −20°C. The identity of the compound was corroborated by MS/MS fragmentation analysis and NMR (Fig. S2 to S22 and Table S5).

### NMR spectroscopy.

Measurements of ^1^H, ^13^C, distortionless enhancement by polarization transfer (DEPT) 135, correlation spectroscopy (COSY), heteronuclear single quantum coherence (HSQC), and heteronuclear multiple-bond correlation (HMBC) spectra were accomplished using an Avance III HD 600-MHz NMR spectrometer (^1^H, 600.05 MHz; ^13^C, 150.88 MHz; Bruker BioSpin GmbH, Ettlingen, Germany). COSY, total correlation spectroscopy (TOCSY), and nuclear Overhauser effect spectroscopy (NOESY) spectra were recorded at 298 K on an Avance Neo 700-MHz NMR spectrometer equipped with a 5-mm CryoProbe Prodigy TCI (^1^H, ^15^N, ^13^C Z-GRD; ^1^H, 700.28 MHz; ^13^C, 176.09 MHz; Bruker BioSpin GmbH, Ettlingen, Germany) with H_2_O suppression. All measurements were carried out using D_2_O as solvent. Chemical shifts are given in parts per million (ppm). ^1^H spectra were referenced to the residual solvent signal (δ = 4.79 ppm). For ^13^C measurements 3-(trimethylsilyl)propionic-2,2,3,3-d_4_ acid sodium salt (TSPA, δ = 1.7 ppm) was used as an external standard. For a better resolution of the correlation signals, HSQC and HMBC spectra were additionally acquired using nonuniform sampling (NUS). Analysis of NMR spectra was achieved using the software TopSpin 3.6.0 (Bruker BioSpin GmbH, Ettlingen, Germany).

### Data availability.

The 16S rRNA gene sequences of the clinical isolates used in this study were deposited in GenBank (accession numbers OP737540 to OP737605; Table S2).
