# Supplementary material for: Antimicrobial Activity of Ceftazidime-Avibactam, Ceftolozane-Tazobactam, Cefiderocol, and Novel Darobactin Analogs against Multidrug-Resistant Pseudomonas aeruginosa Isolates from Pediatric and Adolescent Cystic Fibrosis Patients
Source: Microbiol Spectr. 2023 Jan 24;11(1):e04437-22. doi: 10.1128/spectrum.04437-22 (PMC9927382; doi:10.1128/spectrum.04437-22)
Supplement: Supplemental file 1 — Supplemental material. Download spectrum.04437-22-s0001.pdf, PDF file, 2.2 MB [file spectrum.04437-22-s0001.pdf]

Antimicrobial activity of ceftazidime-avibactam, ceftolozane-tazobactam, cefiderocol and novel darobactin analogs against multi-drug-resistant *Pseudomonas aeruginosa* isolates from pediatric and adolescent cystic fibrosis patients

Michael Marner[1,2,#], Laura Kolberg[3,#], Julia Horst[3,4,5], Nils Böhringer[2,6], Johannes Hübner[3,7], Dewa M. Kresna[2], Yang Liu[2], Ute Mettal[2], Lei Wang[2], Melanie Meyer-Bühn[3], Sanja Mihajlovic[1], Matthias Kappler[3], Till F. Schäberle[1,2,6 #], Ulrich von Both[3,5 #]

[1] Fraunhofer Institute for Molecular Biology and Applied Ecology (IME), Branch for Bioresources, 35392 Giessen, Germany.
[2] Justus-Liebig-University of Giessen, 35392 Giessen, Germany.
[3] Department of Pediatrics, Dr. von Hauner Children's Hospital, University Hospital, LMU Munich, Germany.
[4] Institute for Medical Information Processing, Biometry, and Epidemiology – IBE, LMU Munich, Germany.
[5] Pettenkofer School of Public Health, Munich, Germany.
[6] German Center for Infection Research (DZIF), Partner Site Giessen-Marburg-Langen, Germany.
[7] German Center for Infection Research (DZIF), Partner Site Munich, Germany.

[#] Authors contributed equally

Correspondence:

Till F. Schäberle - ORCID: 0000-0001-9947-8079; Phone: +49 641 97219140; Email: till.f.schaeberle@agrar.uni-giessen.de , till.schaeberle@ime.fraunhofer.de.

Ulrich von Both - ORCID: 0000-0001-8411-1071; Phone: +49 89 440052817; Email: Ulrich.von.both@med.lmu.de

Table of Contents

Table S1 Patient's demographic and clinical data p. 4

Table S2 Strain information: ID, sampling date, resistance profile and MRGN classification p. 5-6

Table S3 Minimum inhibitory concentration (MIC) distribution for ceftazidime-avibactam (CZA), ceftolozane-tazobactam (C/T), and cefiderocol (FDC) p. 7

Table S4 Distribution of susceptibility patterns to ceftazidime-avibactam (CZA), ceftolozane-tazobactam (C/T), and cefiderocol (FDC) p. 8

Figure S1 MS/MS fragmentation signature of darobactin B9 p. 9

NMR data of darobactin B9:

Table S5 ^{1}H (600 MHz) and ^{13}C (150 MHz) NMR data; correlation data from HMBC, COSY, TOCSY, and NOESY experiments p. 10-11

Figure S2: Structure of darobactin B9, COSY, TOCSY, and key HMBC correlations of darobactin B9 p. 12

Figure S3 Comparison of the ^{1}H-NMR spectra of darobactin B9 and darobactin B p. 13

Figure S4 Comparison of the ^{13}C-NMR spectra of darobactin B9 and darobactin B p. 14

Figure S5 ^{1}H-NMR spectrum of darobactin B9 (D2O, 600 MHz) p. 15

Figure S6 ^{1}H-NMR spectrum of darobactin B9 (D_2O, 600 MHz). Close-up in the range of 8.30 - 6.00 ppm p. 16

Figure S7 ^{1}H-NMR spectrum of darobactin B9 (D_2O, 600 MHz). Close-up in the range of 4.90 - 2.60 ppm. p. 17

Figure S8 ^{1}H-NMR spectrum of darobactin B9 (D_2O, 600 MHz). Close-up in the range of 2.25 – 0.65 ppm p. 18

Figure S9 ^{1}H-NMR spectrum of darobactin B9 (D_2O, 700 MHz). p. 19

Figure S10 ^{1}H-NMR spectrum of darobactin B9 (D_2O, 700 MHz). Close-up in the range of 8.30 – 6.10 ppm p. 20

Figure S11 ^{1}H-NMR spectrum of darobactin B9 (D_2O, 700 MHz). Close-up in the range of 4.90 – 2.60 ppm. p. 21

Figure S12 ^{1}H-NMR spectrum of darobactin B9 (D_2O, 700 MHz). Close-up in the range of 2.30 – 0.60 ppm. p. 22

Figure S13 ^{13}C-NMR spectrum of darobactin B9 (D_2O, 150 MHz). For the measurement 3-(trimethylsilyl)propionic-2,2,3,3-d_4 acid sodium salt (TSPA) was used as external standard. The multipletts indicate residual TFA. p. 23

Figure S14 DEPT 135 spectrum of darobactin B9 (D_2O, 150 MHz). For the measurement 3-(trimethylsilyl)propionic-2,2,3,3-d_4 acid sodium salt (TSPA) was used as external standard p. 24

Figure S15 COSY spectrum of darobactin B9 (D_2O, 600 MHz) p. 25

Figure S16 COSY spectrum of darobactin B9 (D_2O, 700 MHz), measured with H_2O suppression. p. 26

Figure S17 TOCSY spectrum of darobactin B9 (D_2O, 700 MHz), measured with H_2O suppression p. 27

Figure S18 NOESY spectrum of darobactin B9 (D_2O, 700 MHz), measured with H_2O suppression. p. 28

Figure S19 HSQC spectrum of darobactin B9 (D_2O, 600 MHz) p. 29

Figure S20 HSQC spectrum of darobactin B9 (D_2O, 600 MHz), measured with non-uniform sampling. p. 30

Figure S21 HMBC spectrum of darobactin B9 (D_2O, 600 MHz) p. 31

Figure S22 HMBC spectrum of darobactin B9 (D_2O, 600 MHz), measured with non-uniform sampling. p. 32

Table S1. Demographic and clinical data collected on each of 35 patients

Patient Number	Sex	Number of *P. aeruginosa* isolates collected	Homozygosity or heterozygosity for the delta F508 mutation	Age in years at first P. aeruginosa detection	Age in years at first MRGN P. aeruginosa detection
Pat01	female	1	other mutation	unknown	unknown
Pat02	male	2	heterozygote	7	unknown
Pat03	male	4	heterozygote	14	unknown
Pat04	female	1	homozygote	5	unknown
Pat05	male	3	heterozygote	11	11
Pat06	male	1	other mutation	16	18
Pat07	male	2	heterozygote	2	2
Pat08	female	1	unknown	unknown	unknown
Pat09	female	1	homozygote	14	unknown
Pat10	female	4	heterozygote	6	unknown
Pat11	female	2	homozygote	22	unknown
Pat12	male	4	other mutation	17	17
Pat13	male	1	homozygote	6	9
Pat14	female	1	other mutation	10	unknown
Pat15	female	1	unknown	unknown	unknown
Pat16	female	8	heterozygote	6	unknown
Pat17	male	7	heterozygote	0	unknown
Pat18	female	1	homozygote	7	11
Pat19	female	1	homozygote	16	unknown
Pat20	female	1	homozygote	16	unknown
Pat21	male	2	unknown	unknown	unknown
Pat22	female	1	homozygote	1	unknown
Pat23	female	2	homozygote	8	unknown
Pat24	male	4	heterozygote	12	unknown
Pat25	female	1	homozygote	18	18
Pat26	male	2	homozygote	unknown	unknown
Pat27	female	2	other mutation	7	unknown
Pat28	male	1	homozygote	3	unknown
Pat29	female	1	other mutation	10	unknown
Pat30	male	1	homozygote	9	unknown
Pat31	female	1	other mutation	8	unknown
Pat32	female	1	homozygote	0	unknown
Pat33	male	1	homozygote	2	unknown
Pat34	female	2	homozygote	13	13
Pat35	female	1	heterozygote	5	unknown

Table S2. Comparison between original and new resistance profile and the resulting MRGN[a] classification for each isolate included in the analysis

MRGN-isolate	Patient-ID	Genbank accession number	Sampling date	Original MRGN classification	Original resistance profile									new MRGN classification								
					TZP	PIP	CAZ	FEP	CIP	MEM	IPM	TOB	CST		PIP	CAZ	FEP	CIP	MEM	IPM	TOB	CST
Isolate 1	Pat01	OP737540	15.09.2006	3-MRGN	R	n.a.	R	n.a.	I	R	n.a.	S	S	4-MRGN	R	R	R	R	R	R	S	S
Isolate 2	Pat02	OP737541	10.05.2007	3-MRGN	R	n.a.	R	n.a.	S	R	n.a.	R	S	4-MRGN	R	R	R	R	R	R	n.a.	n.a.
Isolate 3	Pat02	OP737542	10.05.2007	3-MRGN	R	n.a.	R	n.a.	S	R	n.a.	R	S	4-MRGN	R	R	R	R	R	R	n.a.	n.a.
Isolate 4	Pat03	OP737543	28.09.2006	3-MRGN	R	n.a.	R	n.a.	S	R	n.a.	R	S	4-MRGN	R	R	R	R	R	R	n.a.	n.a.
Isolate 5	Pat03	OP737544	28.09.2006	3-MRGN	R	n.a.	R	n.a.	I	R	n.a.	S	S	4-MRGN	R	R	R	R	R	R	n.a.	n.a.
Isolate 6	Pat03	OP737545	06.03.2007	3-MRGN	R	n.a.	R	n.a.	S	R	n.a.	S	S	4-MRGN	R	R	R	R	R	R	R	S
Isolate 7	Pat03	OP737546	28.09.2006	3-MRGN	R	n.a.	R	n.a.	S	R	n.a.	S	S	4-MRGN	R	R	R	R	R	R	S	S
Isolate 8	Pat04	OP737547	17.10.2006	3-MRGN	S	n.a.	R	n.a.	R	R	n.a.	R	S	non-MRGN	I	I	I	R	I	R	R	S
Isolate 9	Pat05	OP737548	08.09.2008	3-MRGN	R	n.a.	R	n.a.	S	R	n.a.	S	S	4-MRGN	R	R	R	R	R	R	n.a.	n.a.
Isolate 10	Pat05	OP737549	01.10.2008	3-MRGN	R	n.a.	R	n.a.	S	R	n.a.	S	S	3-MRGN	R	R	R	R	I	R	S	S
Isolate 11	Pat05	OP737550	10.11.2008	3-MRGN	R	n.a.	R	n.a.	S	R	n.a.	S	S	4-MRGN	R	R	R	R	R	R	S	S
Isolate 12	Pat06	OP737551	23.12.2008	3-MRGN	R	n.a.	R	n.a.	S	R	n.a.	R	S	non-MRGN	I	I	R	I	S	I	n.a.	n.a.
Isolate 13	Pat07	OP737552	10.06.2010	4-MRGN	R	n.a.	R	n.a.	R	R	n.a.	S	S	4-MRGN	R	R	R	R	R	R	R	n.a.
Isolate 14	Pat07	OP737553	21.12.2011	3-MRGN	R	n.a.	R	n.a.	R	S	n.a.	S	R	non-MRGN	R	I	I	R	S	I	S	S
Isolate 15	Pat08	OP737554	19.11.2010	4-MRGN	R	n.a.	R	n.a.	R	R	n.a.	R	S	4-MRGN	R	R	R	R	R	R	n.a.	n.a.
Isolate 16	Pat09	OP737555	31.05.2007	3-MRGN	R	n.a.	R	n.a.	S	R	n.a.	S	S	4-MRGN	R	R	R	R	R	R	S	S
Isolate 17	Pat10	OP737556	29.08.2013	3-MRGN	R	n.a.	R	n.a.	I	R	n.a.	R	S	4-MRGN	R	R	R	R	R	R	n.a.	n.a.
Isolate 18	Pat10	OP737557	12.10.2006	4-MRGN	R	n.a.	R	n.a.	R	R	n.a.	R	S	4-MRGN	R	R	R	R	R	R	n.a.	n.a.
Isolate 19	Pat10	OP737558	29.08.2013	4-MRGN	R	n.a.	R	n.a.	R	R	n.a.	R	S	4-MRGN	R	R	R	R	R	R	n.a.	n.a.
Isolate 20	Pat10	OP737559	24.05.2007	3-MRGN	R	n.a.	R	n.a.	S	R	n.a.	R	S	4-MRGN	R	R	R	R	R	R	n.a.	n.a.
Isolate 21	Pat11	OP737560	09.11.2006	3-MRGN	R	n.a.	R	n.a.	S	R	n.a.	S	S	4-MRGN	R	R	R	R	R	R	n.a.	n.a.
Isolate 22	Pat11	OP737561	02.02.2007	3-MRGN	R	n.a.	R	n.a.	I	R	n.a.	S	S	4-MRGN	R	R	R	R	R	R	S	S
Isolate 23	Pat12	OP737562	16.04.2008	3-MRGN	R	n.a.	R	n.a.	S	R	n.a.	R	S	4-MRGN	R	R	R	R	R	R	n.a.	S
Isolate 24	Pat12	OP737563	25.03.2008	3-MRGN	R	n.a.	R	n.a.	S	R	n.a.	R	S	3-MRGN	R	R	R	I	R	R	R	S
Isolate 25	Pat12	OP737564	16.04.2008	3-MRGN	R	n.a.	R	n.a.	S	R	n.a.	R	S	3-MRGN	R	R	R	I	R	R	R	S
Isolate 26	Pat12	OP737565	04.03.2009	3-MRGN	R	n.a.	R	n.a.	S	R	n.a.	I	S	4-MRGN	R	R	R	R	R	R	R	S
Isolate 27	Pat13	OP737566	22.09.2017	4-MRGN	R	R	R	R	R	R	n.a.	S	S	non-MRGN	I	I	I	R	S	I	S	S
Isolate 28	Pat14	OP737567	17.10.2018	3-MRGN	R	R	R	R	R	S	S	S	S	non-MRGN	R	R	R	I	S	I	S	S
Isolate 29	Pat15	OP737568	25.07.2007	3-MRGN	R	n.a.	R	n.a.	S	R	n.a.	S	S	3-MRGN	R	R	R	I	R	R	S	S
Isolate 30	Pat16	OP737569	25.03.2010	4-MRGN	R	n.a.	R	n.a.	R	R	n.a.	R	S	4-MRGN	R	R	R	R	R	R	R	S
Isolate 31	Pat16	OP737570	16.04.2009	3-MRGN	R	n.a.	R	n.a.	S	R	n.a.	S	S	4-MRGN	R	R	R	R	R	R	S	S
Isolate 32	Pat16	OP737571	26.10.2010	3-MRGN	R	n.a.	R	n.a.	I	R	n.a.	S	S	4-MRGN	R	R	R	R	R	R	S	S
Isolate 33	Pat16	OP737572	19.12.2007	4-MRGN	R	n.a.	R	n.a.	R	R	n.a.	S	S	4-MRGN	R	R	R	R	R	R	S	S
Isolate 34	Pat16	OP737573	01.07.2009	4-MRGN	R	n.a.	R	n.a.	R	R	n.a.	S	S	4-MRGN	R	R	R	R	R	R	S	S
Isolate 35	Pat16	OP737574	14.01.2010	4-MRGN	R	n.a.	R	n.a.	R	R	n.a.	S	S	4-MRGN	R	R	R	R	R	R	S	S
Isolate 36	Pat16	OP737575	12.05.2010	4-MRGN	R	n.a.	R	n.a.	R	R	n.a.	S	S	4-MRGN	R	R	R	R	R	R	S	S
Isolate 37	Pat17	OP737576	10.04.2013	4-MRGN	R	n.a.	R	n.a.	R	R	n.a.	S	S	4-MRGN	R	R	R	R	R	R	n.a.	n.a.
Isolate 38	Pat17	OP737577	10.08.2016	3-MRGN	R	n.a.	R	R	R	S	n.a.	S	S	non-MRGN	R	I	I	R	I	I	R	S
Isolate 39	Pat17	OP737578	08.09.2016	3-MRGN	R	n.a.	R	S	R	R	n.a.	S	S	non-MRGN	R	R	I	R	I	I	R	S

Isolate 40	Pat17	OP737579	08.03.2018	3-MRGN	R	R	R	R	R	I	S	S	S	non-MRGN	R	I	I	R	I	I	S	S
Isolate 41	Pat17	OP737580	28.06.2017	3-MRGN	S	R	R	R	R	S	n.a.	S	S	non-MRGN	R	I	I	R	I	I	S	S
Isolate 42	Pat17	OP737581	28.06.2017	3-MRGN	R	R	R	S	R	R	n.a.	S	S	3-MRGN	R	R	R	R	I	I	S	S
Isolate 43	Pat17	OP737582	08.03.2018	3-MRGN	R	R	R	S	R	R	R	S	S	4-MRGN	R	R	R	R	R	R	S	S
Isolate 44	Pat18	OP737583	25.03.2009	3-MRGN	R	n.a.	R	n.a.	I	R	n.a.	S	S	non-MRGN	I	I	I	I	S	I	S	S
Isolate 45	Pat19	OP737584	20.09.2006	3-MRGN	R	n.a.	R	n.a.	S	R	n.a.	S	S	4-MRGN	R	R	R	R	R	R	n.a.	n.a.
Isolate 46	Pat20	OP737585	07.03.2007	3-MRGN	R	n.a.	R	n.a.	S	R	n.a.	S	S	4-MRGN	R	R	R	R	R	R	S	S
Isolate 47	Pat21	OP737586	24.10.2017	3-MRGN	R	R	R	R	S	R	n.a.	S	S	non-MRGN	I	I	I	I	S	I	S	S
Isolate 48	Pat21	OP737587	07.03.2017	3-MRGN	R	R	R	R	S	R	n.a.	S	S	3-MRGN	R	R	R	R	S	I	S	S
Isolate 49	Pat22	OP737588	07.02.2007	4-MRGN	R	n.a.	R	n.a.	R	R	n.a.	S	S	4-MRGN	R	R	R	R	R	R	S	S
Isolate 50	Pat23	OP737589	29.01.2014	3-MRGN	R	R	S	R	R	R	n.a.	R	S	4-MRGN	R	R	R	R	R	R	R	S
Isolate 51	Pat23	OP737590	29.01.2014	4-MRGN	R	R	R	R	R	R	n.a.	R	S	4-MRGN	R	R	R	R	R	R	R	S
Isolate 52	Pat24	OP737591	15.03.2007	4-MRGN	R	n.a.	R	n.a.	R	R	n.a.	I	S	4-MRGN	R	R	R	R	R	R	n.a.	n.a.
Isolate 53	Pat24	OP737592	31.08.2006	3-MRGN	R	n.a.	R	n.a.	I	R	n.a.	R	S	non-MRGN	R	I	I	R	I	R	R	S
Isolate 54	Pat25	OP737593	15.02.2007	3-MRGN	R	n.a.	R	n.a.	S	R	n.a.	S	S	3-MRGN	R	R	R	I	R	R	S	S
Isolate 55	Pat26	OP737594	31.03.2017	3-MRGN	R	R	R	R	R	I	n.a.	R	S	non-MRGN	I	I	R	I	S	I	S	S
Isolate 56	Pat26	OP737595	26.05.2017	3-MRGN	R	R	R	R	R	S	n.a.	S	S	non-MRGN	I	R	R	R	S	R	S	S
Isolate 57	Pat27	OP737596	04.04.2007	3-MRGN	R	n.a.	R	n.a.	R	S	n.a.	S	S	3-MRGN	R	R	R	R	I	R	S	S
Isolate 58	Pat28	OP737597	07.09.2006	3-MRGN	R	n.a.	S	n.a.	R	R	n.a.	R	S	non-MRGN	R	I	I	R	I	I	R	S
Isolate 59	Pat29	OP737598	15.11.2006	3-MRGN	R	n.a.	R	n.a.	S	R	n.a.	R	S	non-MRGN	R	R	R	I	S	I	R	S
Isolate 60	Pat30	OP737599	01.09.2006	3-MRGN	R	n.a.	R	n.a.	I	R	n.a.	S	S	4-MRGN	R	R	R	R	R	R	R	S
Isolate 61	Pat31	OP737600	08.09.2006	3-MRGN	R	n.a.	R	n.a.	I	R	n.a.	S	S	3-MRGN	R	R	R	R	I	R	R	S
Isolate 62	Pat32	OP737601	30.04.2015	3-MRGN	R	n.a.	S	n.a.	R	R	n.a.	S	S	4-MRGN	R	R	R	R	R	R	S	S
Isolate 63	Pat33	OP737602	11.02.2013	3-MRGN	R	n.a.	R	n.a.	S	R	n.a.	S	S	4-MRGN	R	R	R	R	R	R	n.a.	n.a.
Isolate 64	Pat34	OP737603	02.11.2018	3-MRGN	S	R	R	R	R	S	S	R	S	non-MRGN	R	R	I	R	S	I	R	S
Isolate 65	Pat34	OP737604	14.06.2017	3-MRGN	R	R	R	R	R	I	R	R	S	3-MRGN	R	R	R	R	S	I	R	S
Isolate 66	Pat35	OP737605	05.02.2007	3-MRGN	R	n.a.	R	n.a.	R	S	n.a.	R	S	non-MRGN	R	I	R	R	S	R	R	S

TZP: piperacillin-tazobactam, PIP: piperacillin, CAZ: ceftazidime, FEP: Cefepime, CIP: Ciprofloxacin, MEM: Meropenem, IPM: Imipenem, TOB: Tobramycin, CST: Colistin, n.a.: not available
[a]MRGN (multidrug-resistant gramnegative bacteria) is classified according to KRINKO as unsusceptible to the lead compounds of 3 (3-MRGN) or 4 (4-MRGN) of the following antibiotic classes: acyloreidopenicillins (piperacillin-tazobactam or piperacillin), third- or fourth-generation cephalosporins (ceftazidime and cefepime), fluoroquinolones (ciprofloxacin) and carbapenems (meropenem and imipenem).

Table S3. Minimum inhibitory concentration (MIC) distribution for ceftazidime-avibactam (CZA), ceftolozane-tazobactam (C/T), and cefiderocol (FDC) for 66 *Pseudomonas aeruginosa* isolates. Breakpoints determined by European Committee on Antimicrobial Susceptibility Testing (EUCAST) are used to define bacterial isolates as resistant or susceptible.

Antimicrobial Agent	Numberof Isolates with indicated MIC (µg/mL)										classification category according to EUCAST criteria	
	≤ 0.25	0.38-1.0	1.5-2.0	3.0-4.0	6.0-8.0	12.0-16.0	24.0-64.0	>96	MIC50 (µg/m	MIC90 (µg/m	Susceptible	Resistant
CZA	0	0	4	16	11	8	12	15	12	256	31	35
C/T	0	2	12	20	7	12	2	11	4	256	34	32
FDC	21	10	15	8	10	2	0	0	1,5	6	46	20

Berechnete % für obige Tabelle

Antimicrobial Agent	% of Isolates with indicated MIC (µg/mL)										% of Isolates per classification category	
	≤ 0.25	0.38-1.0	1.5-2.0	3.0-4.0	6.0-8.0	12.0-16.0	24.0-64.0	>96	MIC50 (µg/m	MIC90 (µg/m	Susceptible	Resistant
CZA	0	0	6,1	24,2	16,7	12,1	18,2	22,7			47	53
C/T	0	3	18,2	30,3	10,6	18,2	3	16,7			51,5	48,5
FDC	31,8	15,2	22,7	21,1	15,2	3	0	0			69,7	30,3

Table S4. Distribution of susceptibility patterns to ceftazidime-avibactam (CZA), ceftolozane-tazobactam (C/T), and cefiderocol (FDC) of clinical *P. aeruginosa* isolates (n = 66).

	total (n=66)		Non-MRGN (n=18)		3-MRGN (n=10)		4MRGN (n=38)	
	n	%	n	%	n	%	n	%
3xR	14	21,2	1	5,6	1	10	12	31,6
1xS	18	27,3	1	5,6	3	30	14	36,8
2xS	9	13,6	2	11,1	0	0	7	18,4
3xS	25	37,9	14	77,7	6	60	5	13,2

MRGN (multidrug-resistant Gram-negative bacteria) is classified according to KRINKO as unsusceptible to the lead compounds of 3 (3-MRGN) or 4 (4-MRGN) of the following antibiotic classes: acylureidopenicillins (piperacillin-tazobactam or piperacillin), third- or fourth-generation cephalosporins (ceftazidime and cefepime), fluoroquinolones (ciprofloxacin) and carbapenems (meropenem and imipenem).

3xR/ 3xS: proportion of isolates that were resistant (R)/ susceptible (S) to all three antibiotics tested (CZA, C/T and FDC)

2xS/ 1xS: proportion of isolates with susceptibility (S) to two or one of the antibiotics tested.

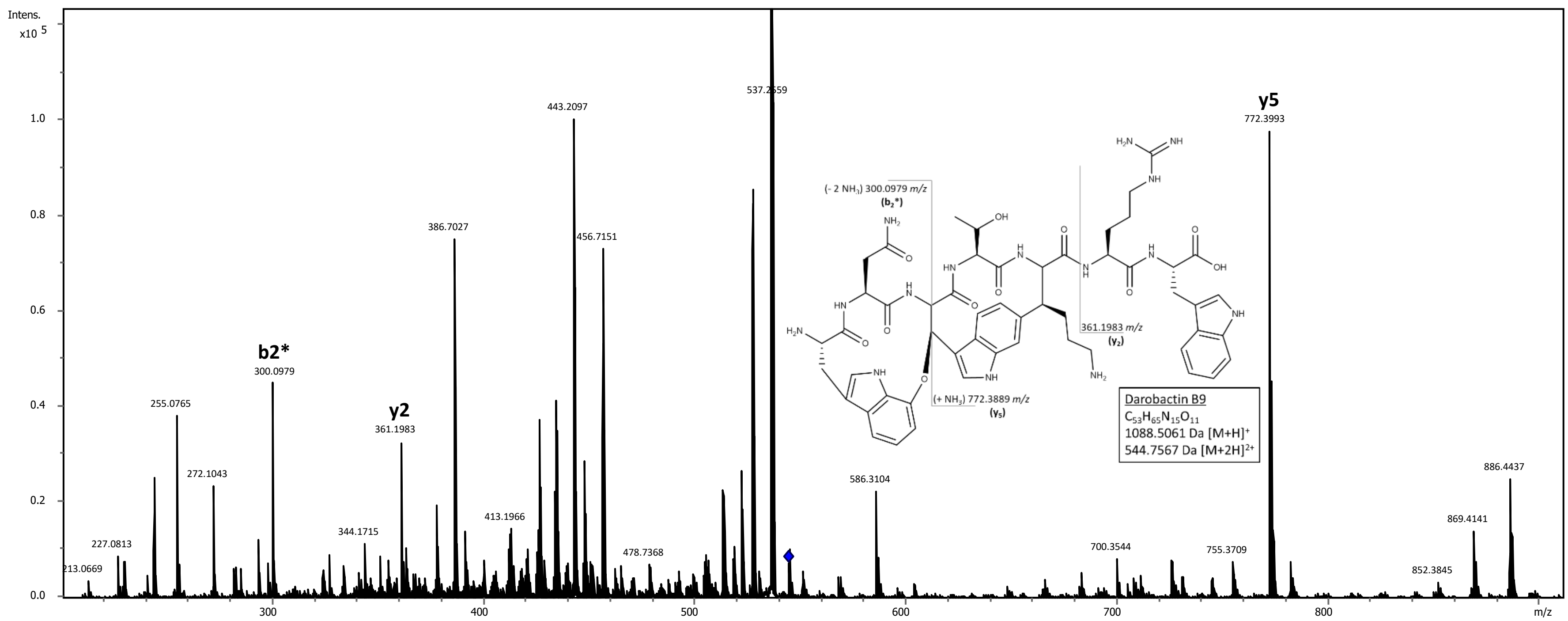

Figure S1. Fragmentation signature of darobactin B9. Major fragment ions observed are OH-W^1N^2 300.0979 *m/z* (Δ 0.7 ppm, b_2*) as well as $W^3T^4K^5R^6W^7$ 772.3889 (Δ 13 ppm, y_5) and R^6W^7 361.1983 (Δ 0.7 ppm, y_2).

118

Table S5: ^{1}H (600 MHz) and ^{13}C (150 MHz) NMR data of Darobactin B9 (D_2O; δ in ppm) alongside correlation data from HMBC, COSY, TOCSY, and NOESY experiments. For ^{13}C measurements 3-(trimethylsilyl) propionic-2,2,3,3-d$_4$ acid sodium salt (TSPA) was used as external standard. The following abbreviations are used in this table: mult.: multiplicity, int.: integral, obs.: obscured. For atom numbering, cf. Figure S 2.

Amino acid	Position	δ_C [ppm], Type	δ_H [ppm][a], mult. (J in Hz), int.	HMBC[b] correlation to position	COSY[b] correlation to position	TOCSY[b], [c] correlation to position	NOESY[b], [c] correlation to position
Trp (N-terminal)	1	59.1, CH	4.03, dd (7.3, 10.9), 1H	2, (11)	2	2	4
	2	30.7, CH_2	3.55, dd (7.2, 13.7), 1H<br>3.307, m, 1H[d]	1, 3, (4), 10, 11	1	1, (4)	7+9, 8
	3	112.6, C_q	/				
	4	129,1, CH	7.35, s, 1H	3, 5 and/or 10, (6)	/	(2)	1
	5	133.5, C_q	/				
	6	149.6, C_q	/				
	7 + 9	118.3, CH;<br>113.5, CH	7.25, pseudo-t (7.8), 2H	5 and/or 10, 6, 7, 9	8	8	2, 17
	8	124.7, CH	7.19, t (7.7), 1H	6, 10	7 + 9	7 + 9	2, 17
	10	133.4, C_q	/				
	11	172.7, C_q	/				
Asn	12	55.2, CH	3.32, m, 1H[d]	(11), 13, 14, (15)	13	13	/
	13	43.3, CH_2	2.16, m, 2H	12, 14, 15	12	12	/
	14	178.2, C_q	/				
	15	173.0, C_q	/				
Trp	16	67.8, CH	4.70, d (8.9), 1H[e]	(15), 17, 26	17	17	/
	17	81.4, CH	6.20, d (8.9), 1H	6, (16), 18, 19	16	16	7+9, 8, 12, 23, 24, (27)
	18	116.1, C_q	/				
	19	128.9, CH	7.85, s, 1H	(17), 18, 20, 25	/	/	/
	20	141.6, C_q	/				
	21	114.9, CH	7.41, s, 1H	32, 23 and/or 25	/	/	31, (32), 33, (35)
	22	137.4, C_q	/				
	23	129.31, CH	6.92, dd (1.0, 8.4), 1H	21, 32, 25	24	24	17, 27, 31, 32, 33, (34)
	24	121.9, CH	7.43, d (8.2), 1H	(18), 20, 22	23	23	17, 32, (27)
	25	129.28, C_q	/				
	26	172.6, C_q	/				
Thr	27	62.6, CH	3.71, d (6.5), 1H	(26), 28, 29, 30	28	28, 29	23, (24)
	28	72.5, CH	3.37, p (obs)[f], 1H	(27), 29, 30	27, 29	27, 29	/
	29	22.7, CH_3	0.78, d (6.4), 3H	27, 28	28	27, 28	/
	30	172.4, C_q	/				

Table S5 (continued):

Amino acid	Position	δ_C [ppm], Type	δ_H [ppm][a], mult. (J in Hz), int.	HMBC[b] correlation to position	COSY[b] correlation to position	TOCSY[b], [c] correlation to position	NOESY[b], [c] correlation to position
Lys	31	64.5, CH	4.08, d (10.6), 1H	(22), (30), 32, 33, 36	32	32, 33, 34, 35	21, 23
	32	52.4, CH	2.94, td (3.5, 11.1), 1H	/	31, 33	31, 33, 34, 35	21, 23, 24
	33	30.0, CH$_2$	1.79, m, 1H; 1.42, m, 1H[g]	/	32	31, 32, 34, 35	21, 23
	34	29.9, CH$_2$	1.73, m, 1H[h]; 1.53, m, 1H	/	35	31, 32, 33, 35	(23)
	35	43.6, CH$_2$	2.73, m, 2H	33 and/or 34	34	31, 32, 33, 34	(21)
	36	175.9, C$_q$	/				
Arg	37	57.8, CH	4.31, dd (5.9, 8.9), 1H	(36), 38, (39), 42	38	38, 39, 40	/
	38	32.4, CH$_2$	1.72, m, 1H[h]; 1.58, m, 1H	(37), (39), (40), (42)	37, 39	37, 39, 40	/
	39	28.8, CH$_2$	1.43, m, 2H[g]	(37), (38), (40)	38, 40	37, 38, 40	/
	40	44.8, CH$_2$	3.06, t (7.4), 2H	38, 39, 41	39	37, 38, 39	/
	41	161.1, C$_q$	/				
	42	177.1, C$_q$	/				
Trp (C-terminal)	43	58.9, CH	4.69, t (6.2), 1H[e]	(42), 44, 45, 53	44	44	/
	44	31.3, CH$_2$	3.41, dd (5.0, 14.8), 1H 3.314, m, 1H[d]	43, 45, (46), 52, 53	43	43, (46)	/
	45	113.9, C$_q$	/				
	46	129.2, CH /	7.31, s, 1H	(44), 45, 47, 52	/	(44)	/
	47	140.7, C$_q$	/				
	48	116.4, CH	7.57, d (8.2), 1H	50, 52	49	49, 50, 51	/
	49	126.5, CH	7.29, m, 1H	51, (47)	48, 50	48, 50, 51	/
	50	123.9, CH	7.21, m, 1H	48, 52	49, 51	48, 49, 51	/
	51	123.1, CH	7.72, d (8.0), 1H	45, 47, 49, (52)	50	48, 49, 50	/
	52	131.7, C$_q$	/				
	53	180.5, C$_q$	/				

[a] The ^{1}H shifts of multiplets were extracted from the HSQC spectrum (600 MHz, 150 MHz; non-uniform sampling). [b] Brackets indicate weak correlation signals. [c] TOCSY and NOESY spectra were recorded at 700 MHz with H_2O suppression. [d] The observed integral for this signal was 3H due to the overlay of the proton signals for H-2, H-12, and H-44. Thus, for each of these positions an integral of 1H was assigned. [e] The observed integral for this signal was 2H due to the overlay of the proton signals for H-16 and H-43. Thus, for each of these positions an integral of 1H was assigned. [f] The pentet (p) for H-28 is partly obscured in the spectrum measured at 600 MHz (Figure S7), but can be observed in the spectrum measured at 700 MHz (Figure S11; J = 6.4 Hz). [g] The observed integral for this signal was 3H due to the overlay of the proton signals for H-33 and H-39. Consequently, for H-33 an integral of 1H was assigned, while for H-39 an integral of 2H was assigned. [h] The observed integral for this signal was 3H due to the overlay of the proton signals for H-34 and H-38 with an impurity. For each of these positions an integral of 1H was assigned. The observed integral for this signal was 3H due to the overlay of the proton signal for H-35 with an impurity. The expected integral for H-35 is 2H.

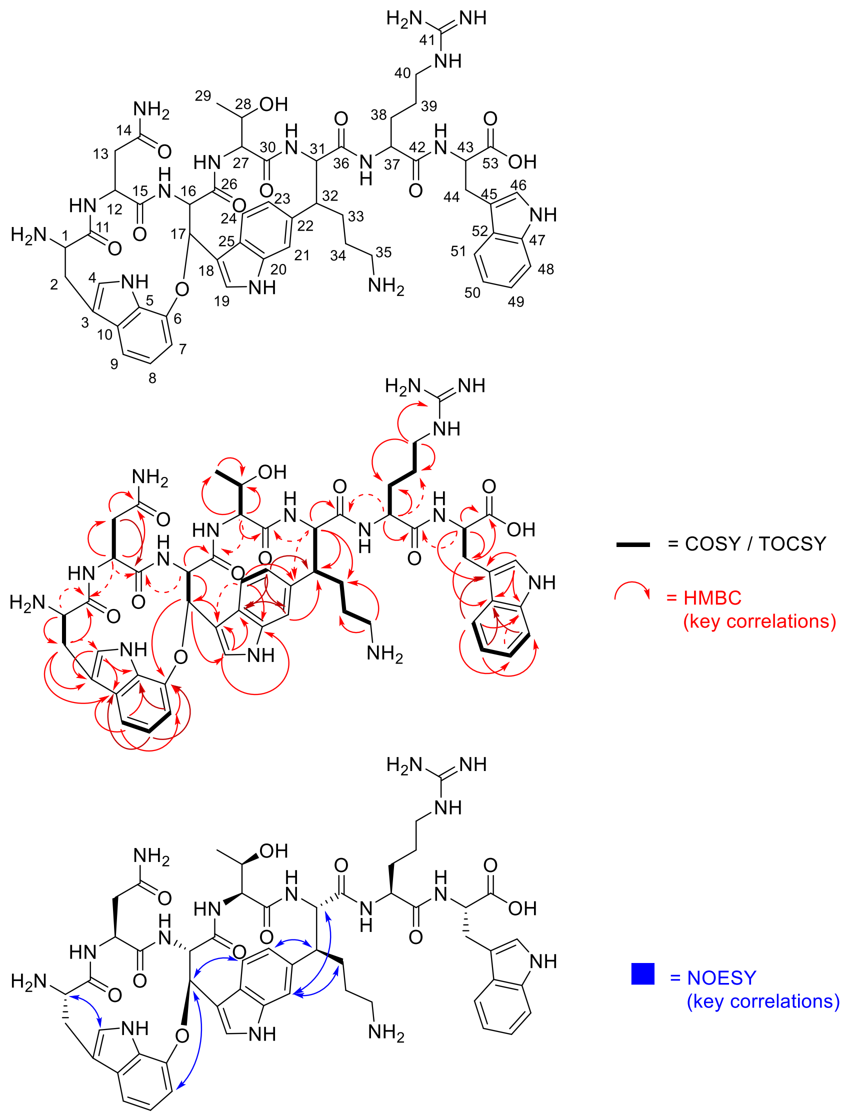

Figure S2: (a) Structure of darobactin B9, including the atom numbering used for NMR structure elucidation. (b) COSY, TOCSY, and key HMBC correlations of darobactin B9. (Dashed arrow indicate weak correlation signals.) (c) Key NOESY correlations of darobactin B9.

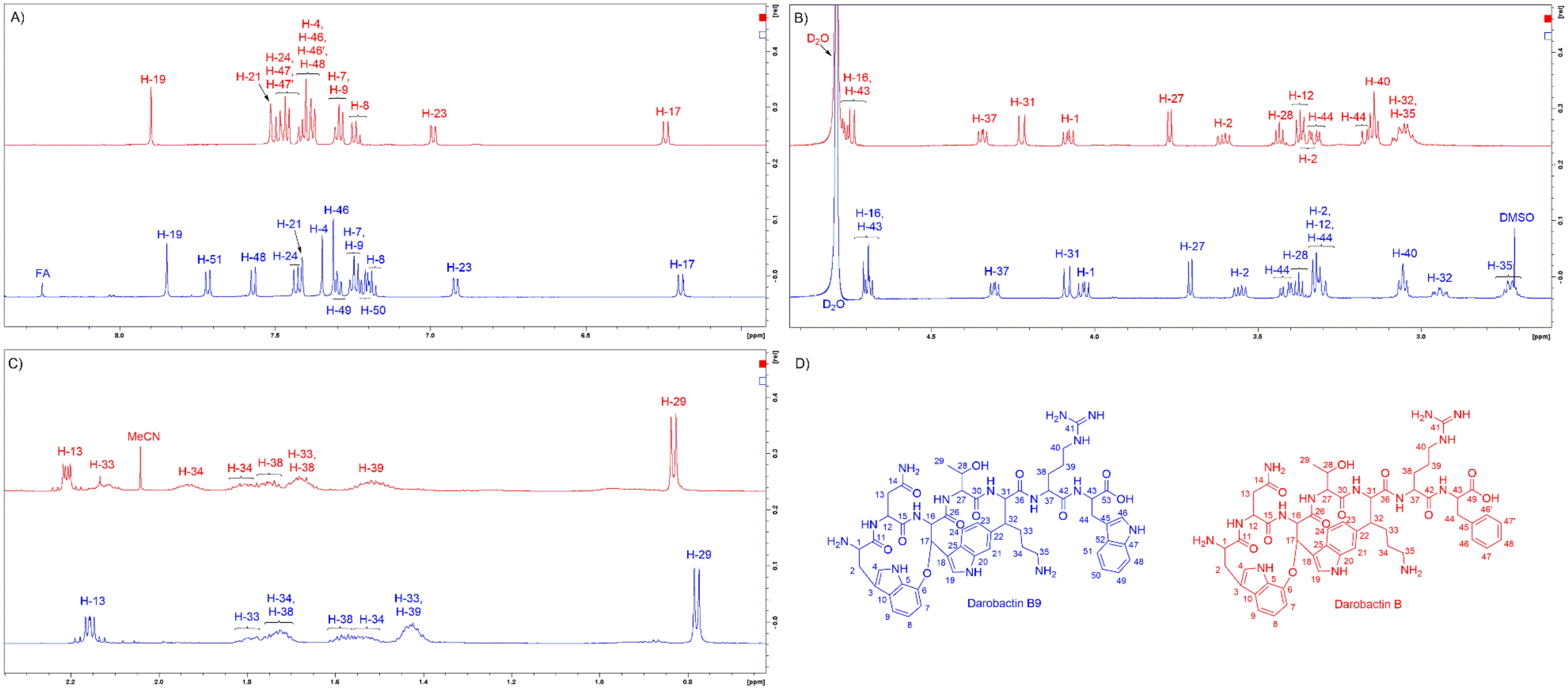

Figure S3: Comparison of the ^{1}H-NMR spectra of darobactin B9 (blue) and darobactin B (red). Both spectra were measured in D_2O at 600 MHz.

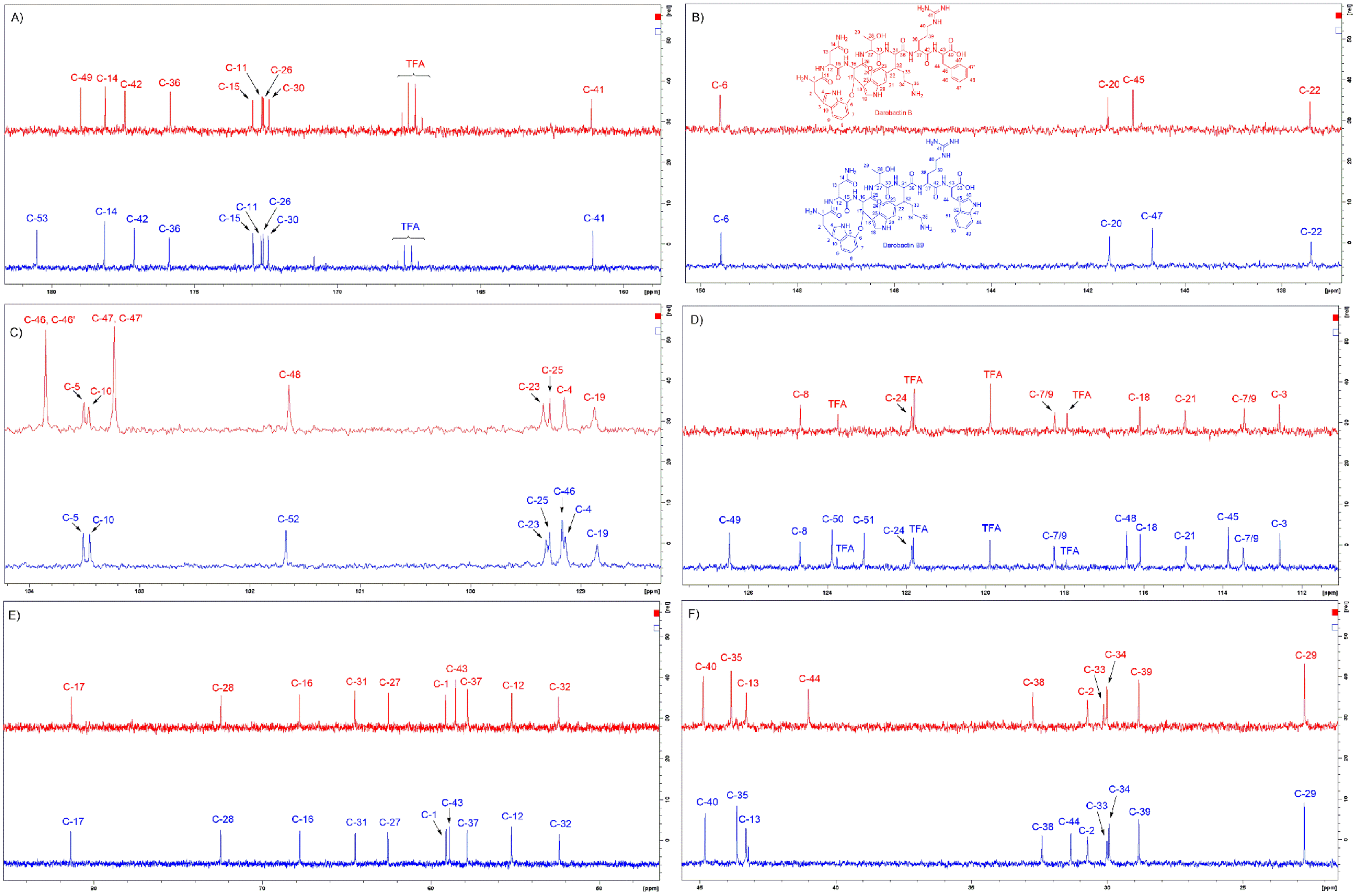

Figure S4: Comparison of the ^{13}C-NMR spectra of darobactin B9 (blue) and darobactin B (red). Both spectra were measured in D_2O at 600 MHz. As external standard 3-(trimethylsilyl)propionic-2,2,3,3-d$_4$ acid sodium salt (TSPA) was used.

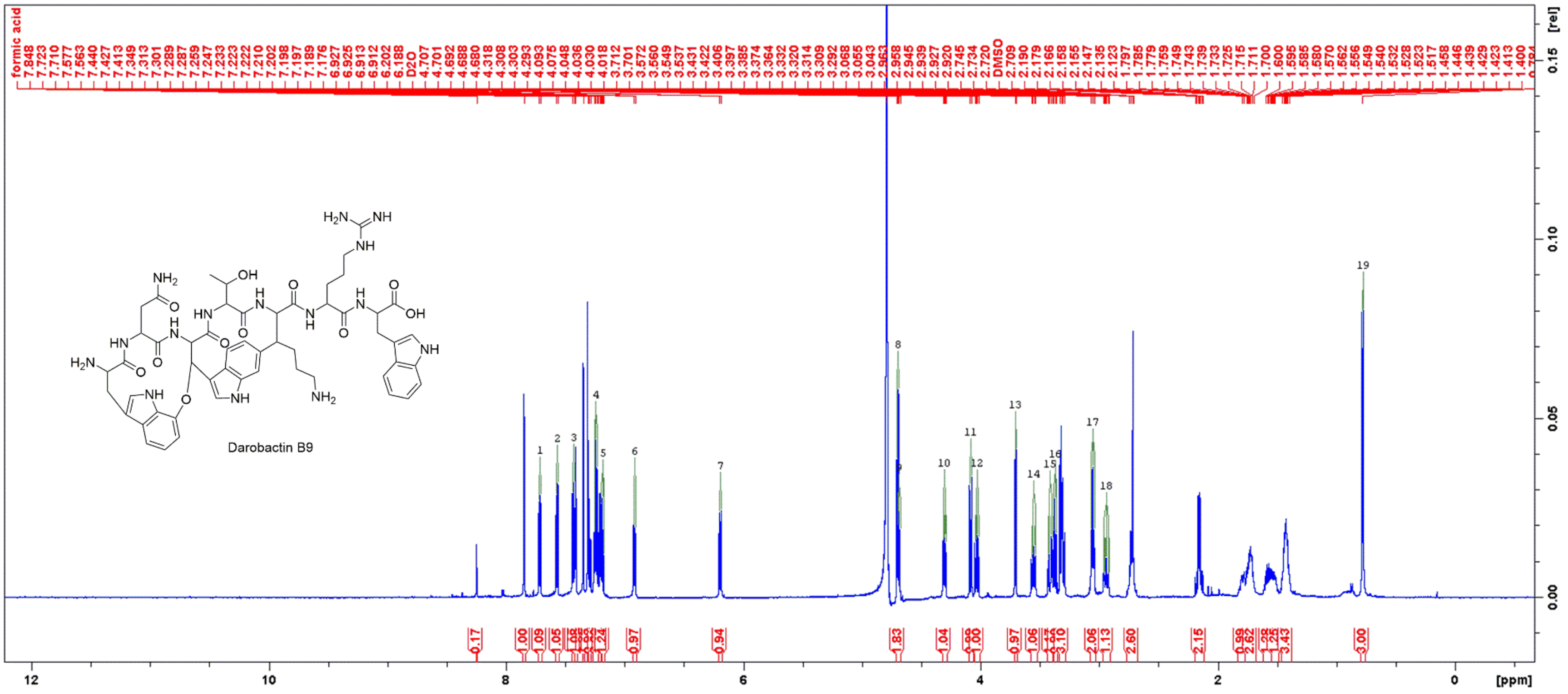

Figure S5: ^{1}H-NMR spectrum of darobactin B9 (D_2O, 600 MHz).

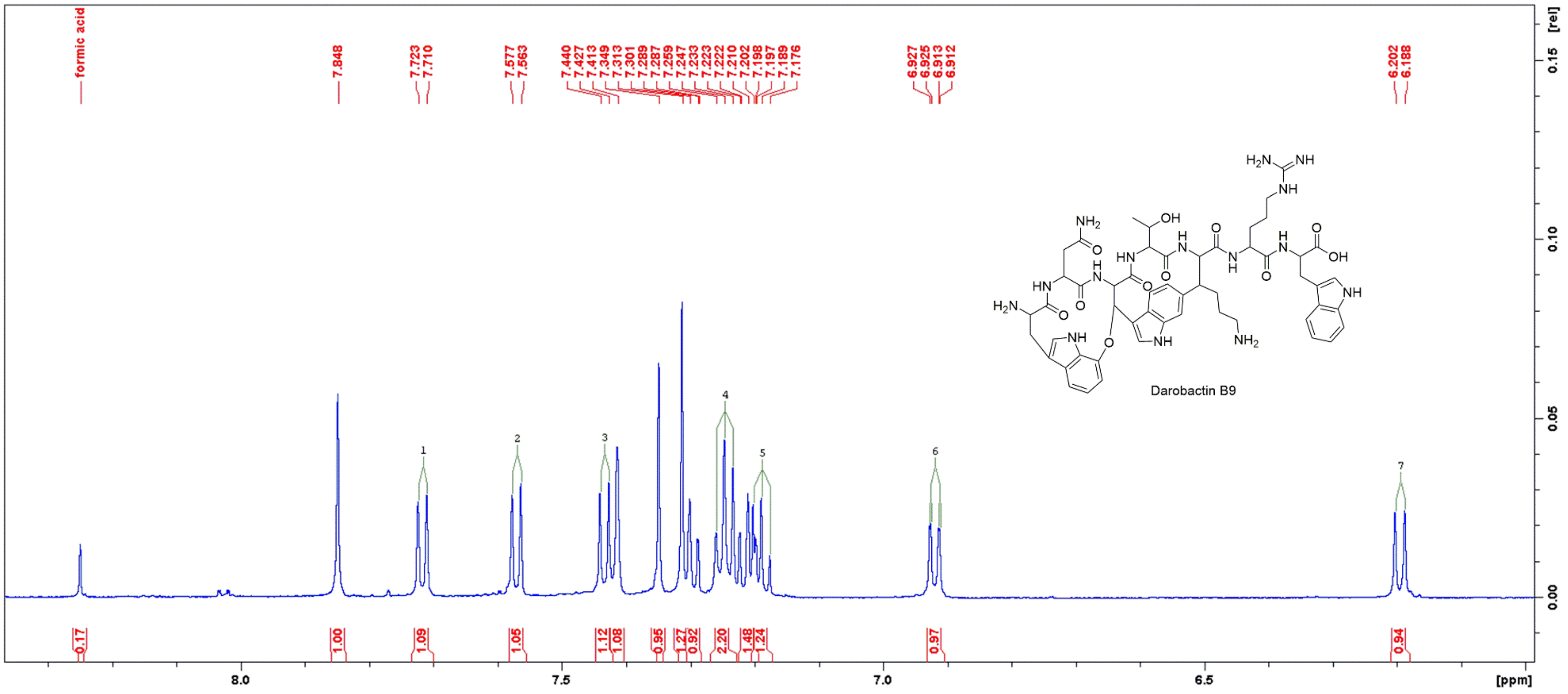

Figure S6: ^{1}H-NMR spectrum of darobactin B9 (D_2O, 600 MHz). Close-up in the range of 8.30 - 6.00 ppm.

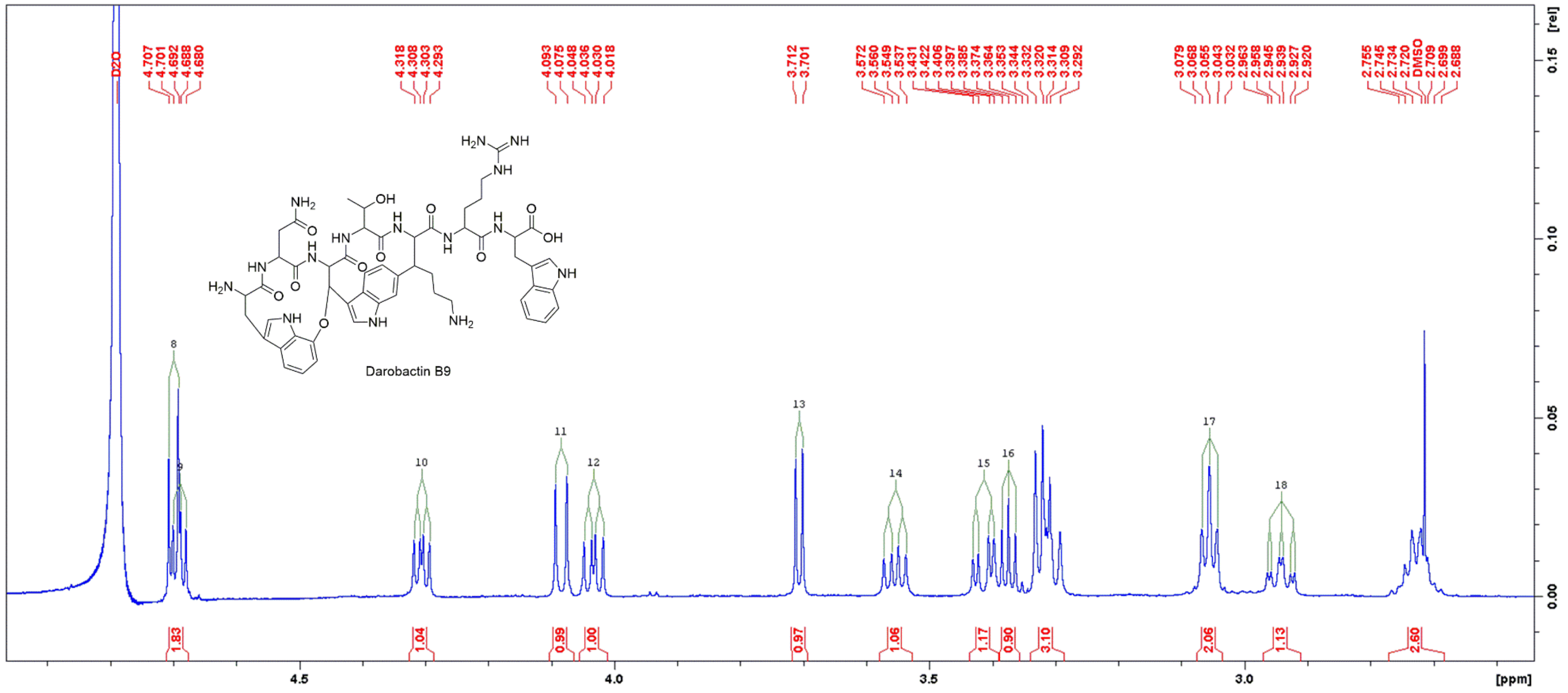

Figure S7: ^{1}H-NMR spectrum of darobactin B9 (D_2O, 600 MHz). Close-up in the range of 4.90 - 2.60 ppm.

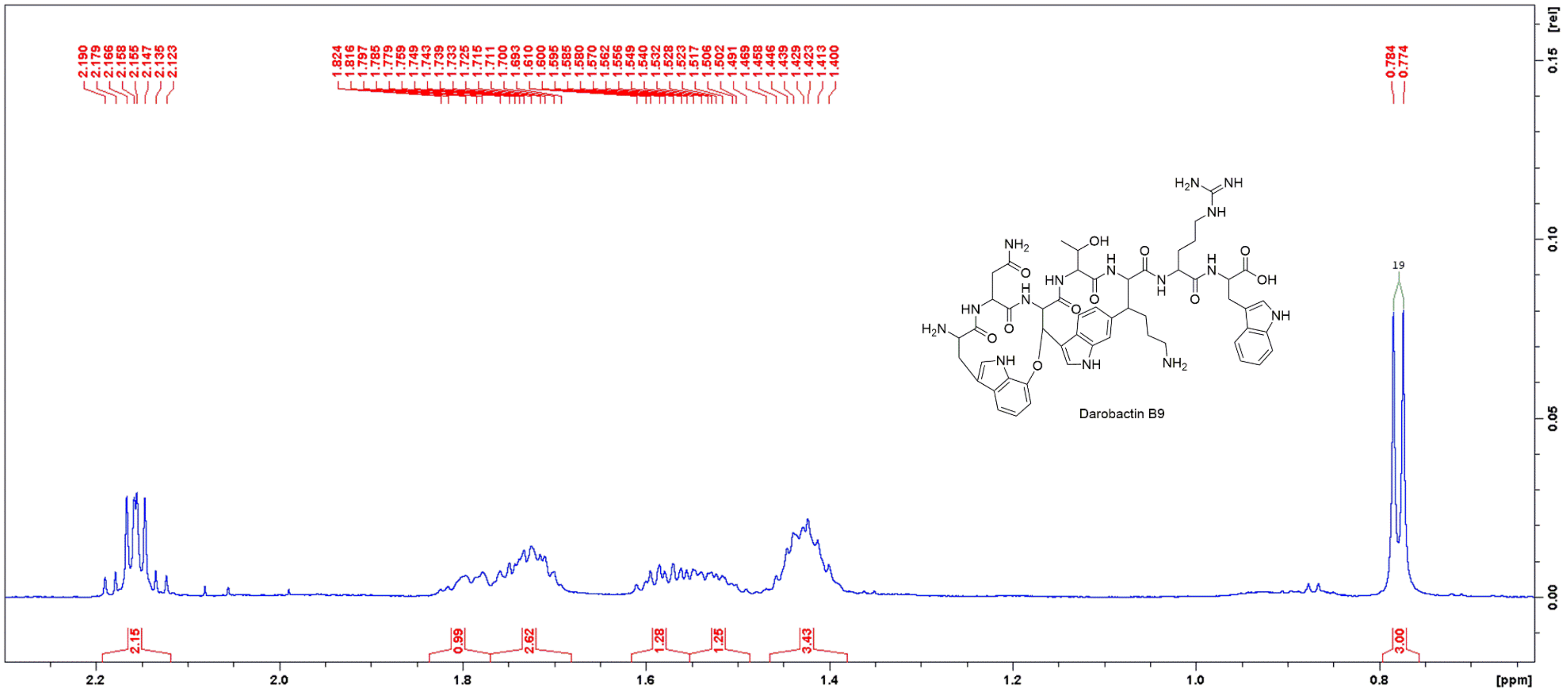

Figure S8: ^{1}H-NMR spectrum of darobactin B9 (D_2O, 600 MHz). Close-up in the range of 2.25 – 0.65 ppm.

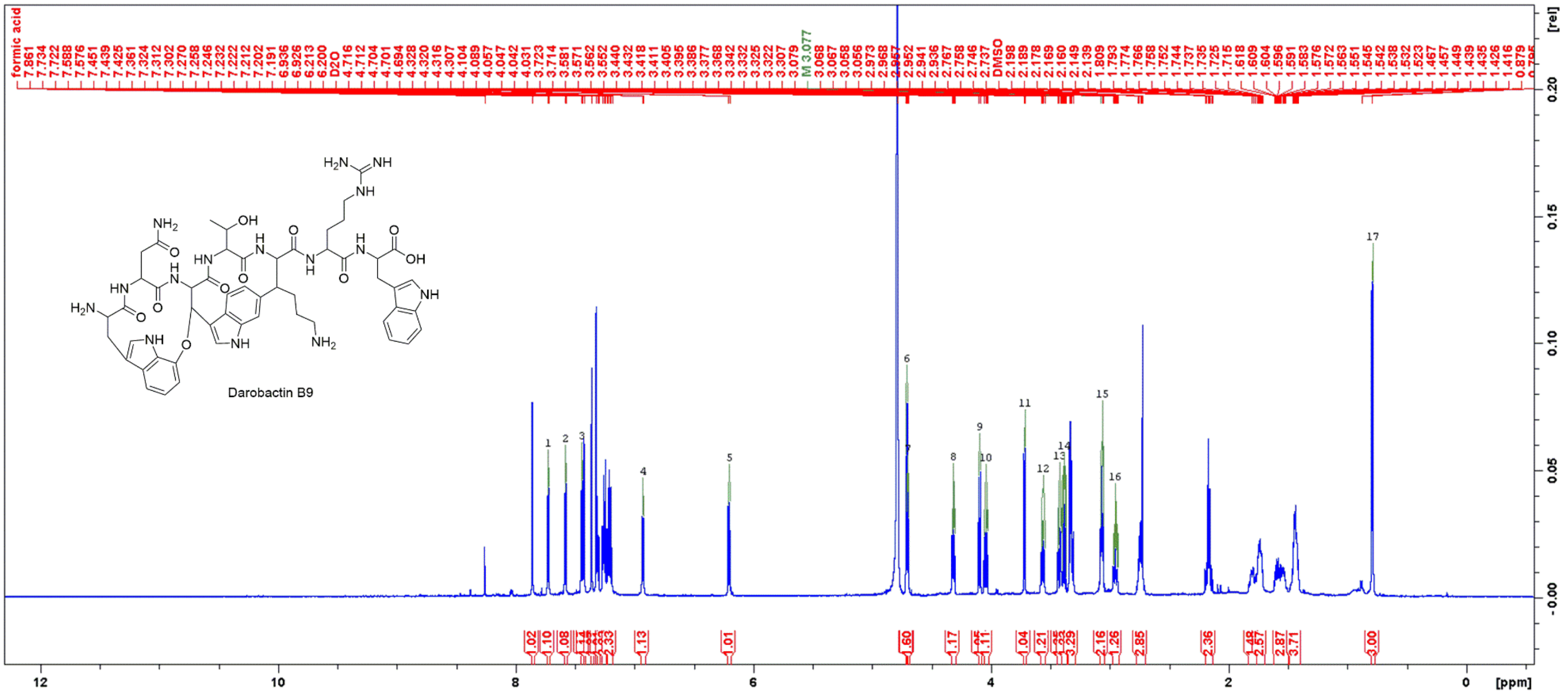

Figure S9: ^{1}H-NMR spectrum of darobactin B9 (D_2O, 700 MHz).

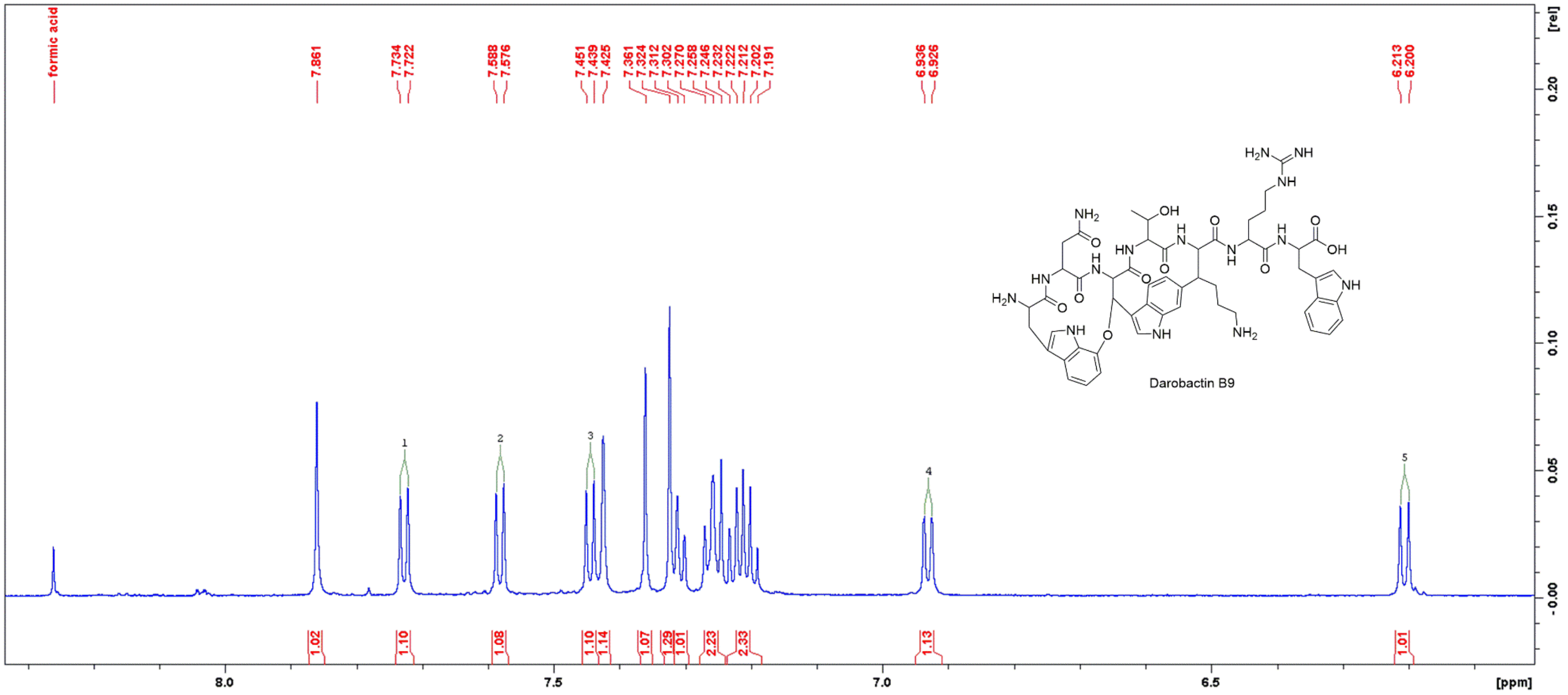

Figure S10: ^{1}H-NMR spectrum of darobactin B9 (D_2O, 700 MHz). Close-up in the range of 8.30 – 6.10 ppm.

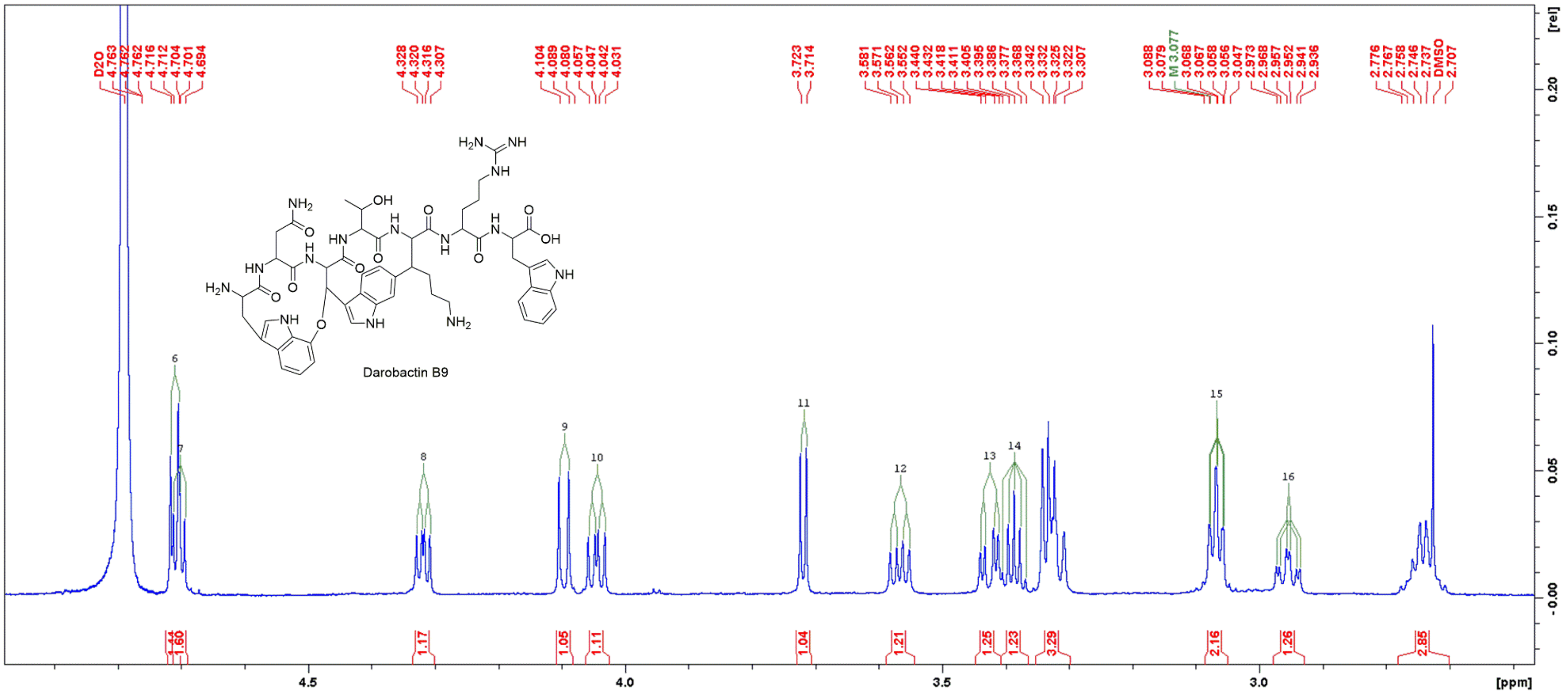

Figure S11: ^{1}H-NMR spectrum of darobactin B9 (D_2O, 700 MHz). Close-up in the range of 4.90 – 2.60 ppm.

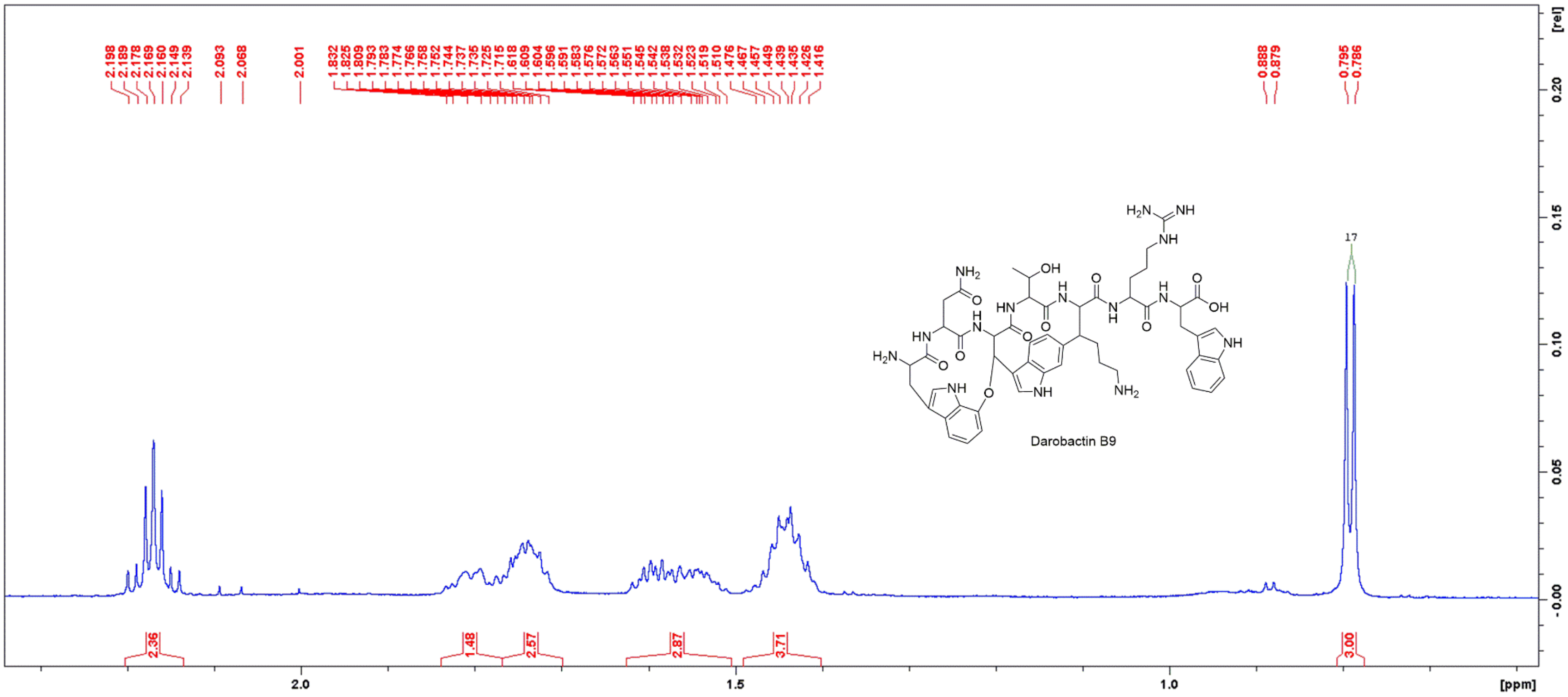

Figure S12: ^{1}H-NMR spectrum of darobactin B9 (D_2O, 700 MHz). Close-up in the range of 2.30 – 0.60 ppm.

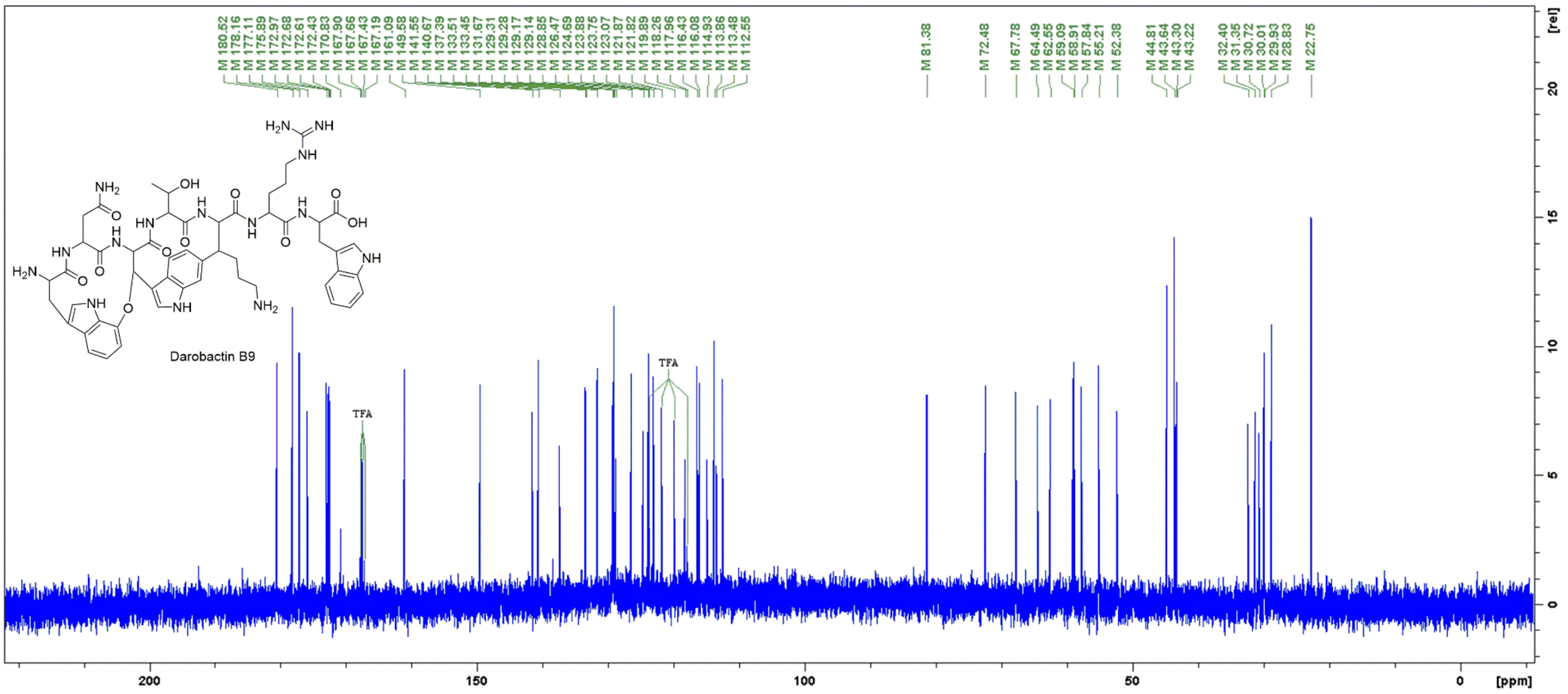

Figure S13: ^{13}C-NMR spectrum of darobactin B9 (D_2O, 150 MHz). For the measurement 3-(trimethylsilyl)propionic-2,2,3,3-d_4 acid sodium salt (TSPA) was used as external standard. The multipletts indicate residual TFA.

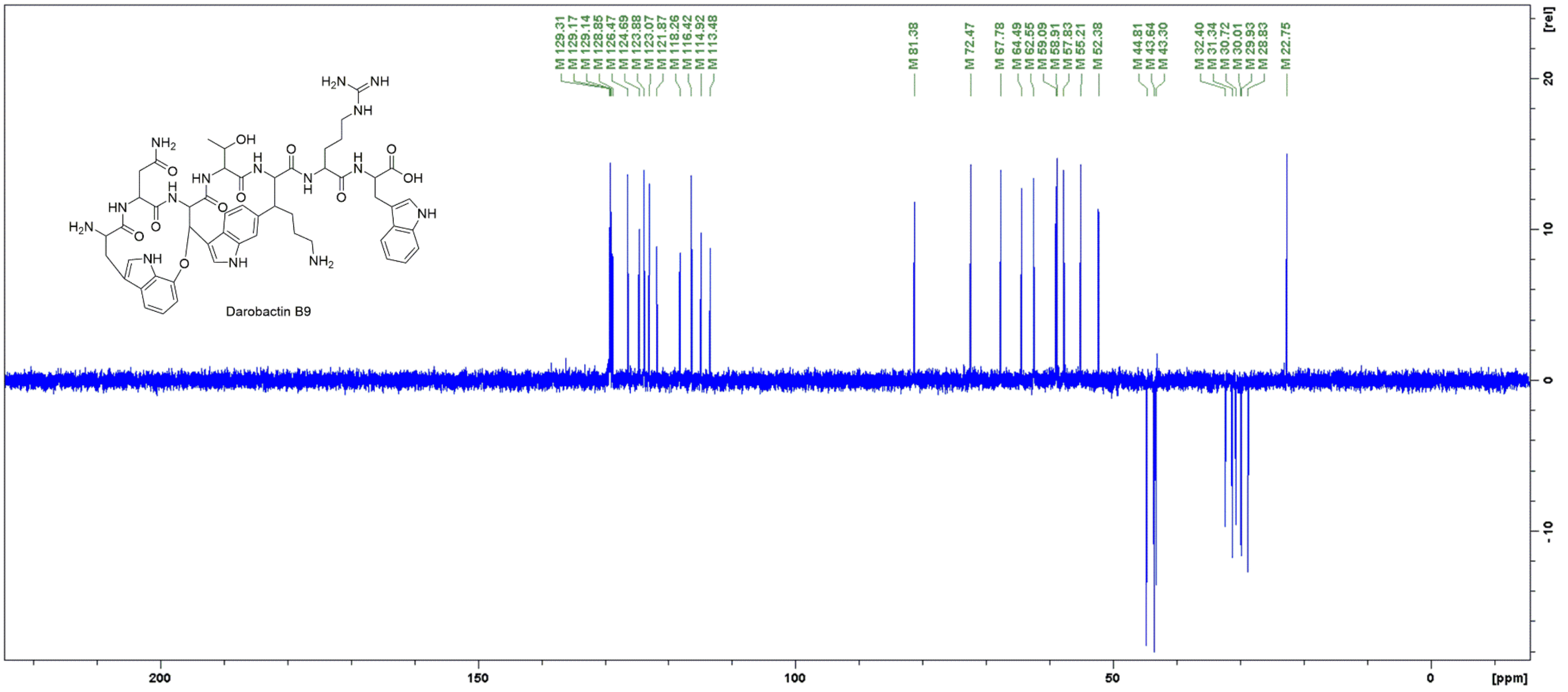

Figure S14: DEPT 135 spectrum of darobactin B9 (D_2O, 150 MHz). For the measurement 3-(trimethylsilyl)propionic-2,2,3,3-d_4 acid sodium salt (TSPA) was used as external standard.

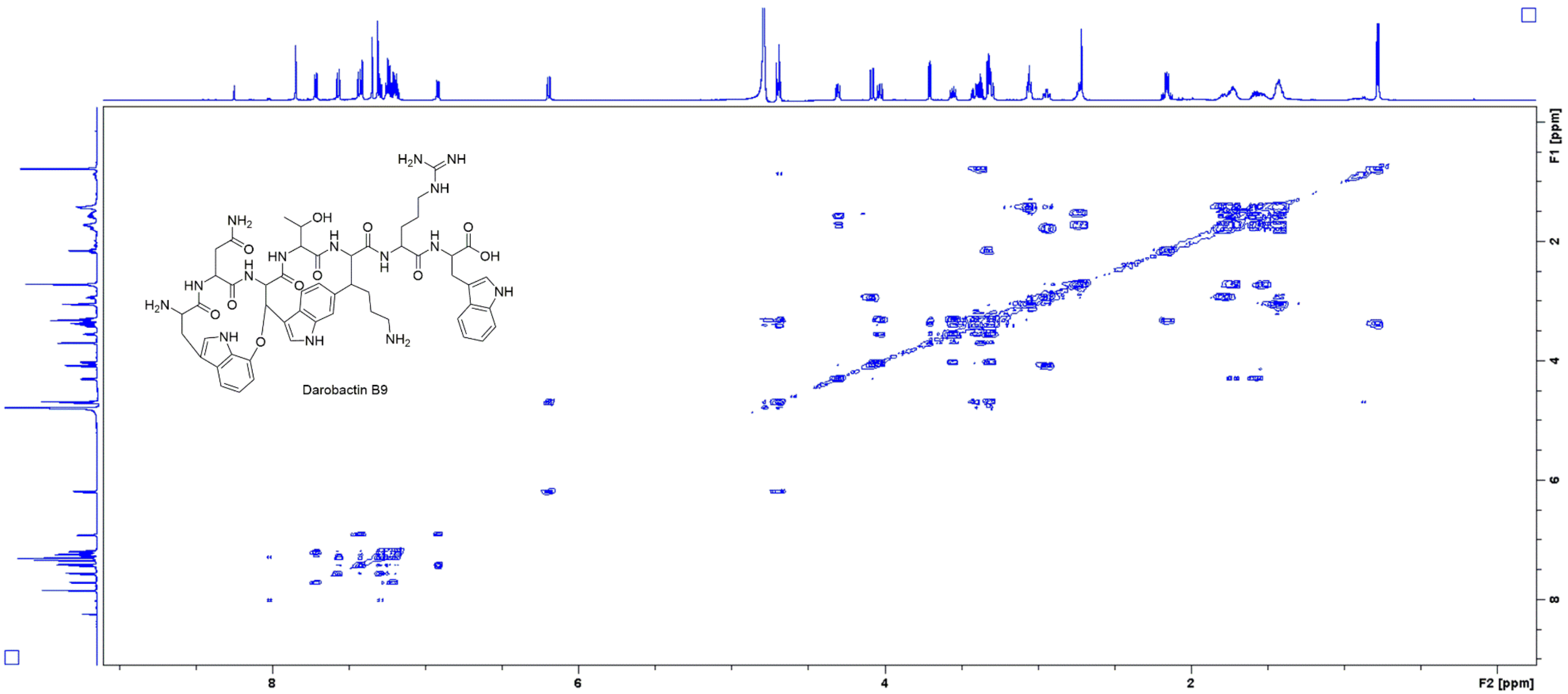

Figure S15: COSY spectrum of darobactin B9 (D_2O, 600 MHz).

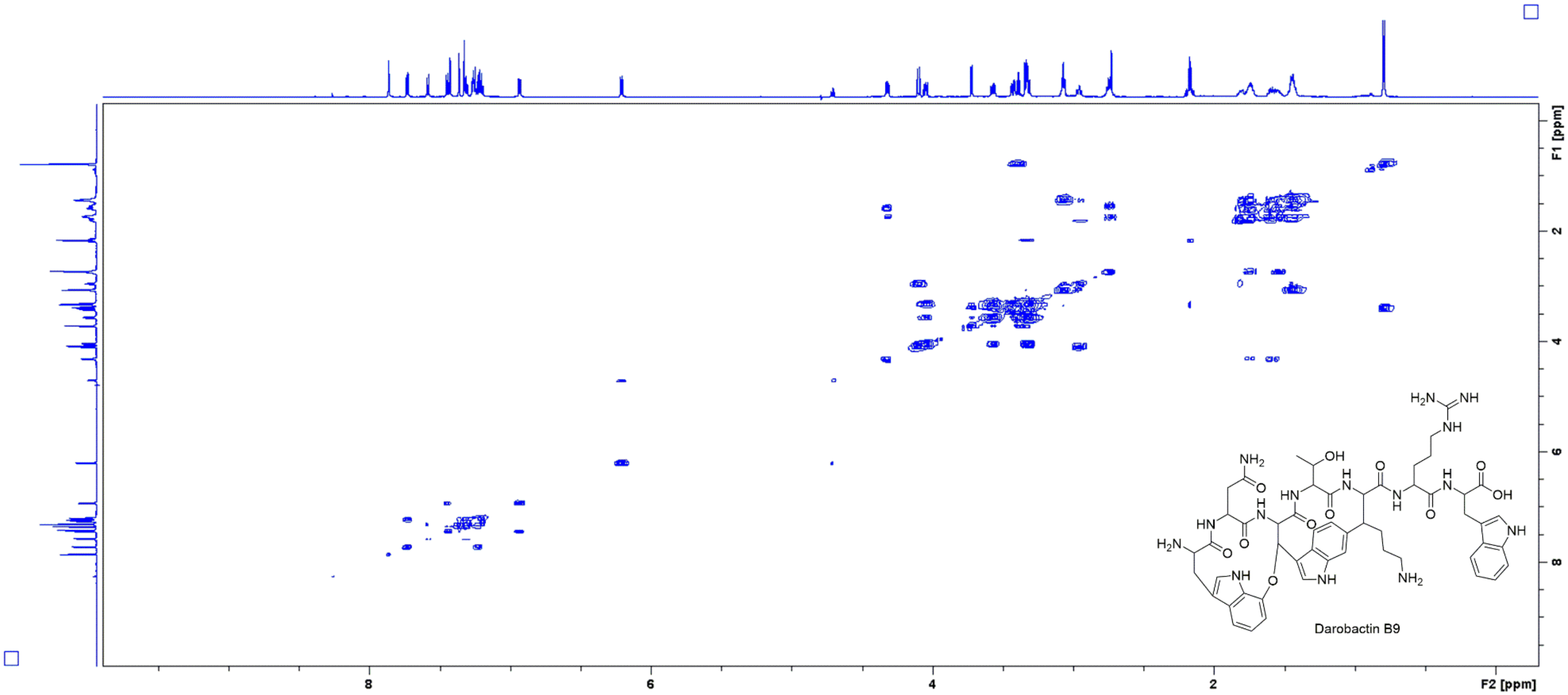

Figure S16: COSY spectrum of darobactin B9 (D_2O, 700 MHz), measured with H_2O suppression.

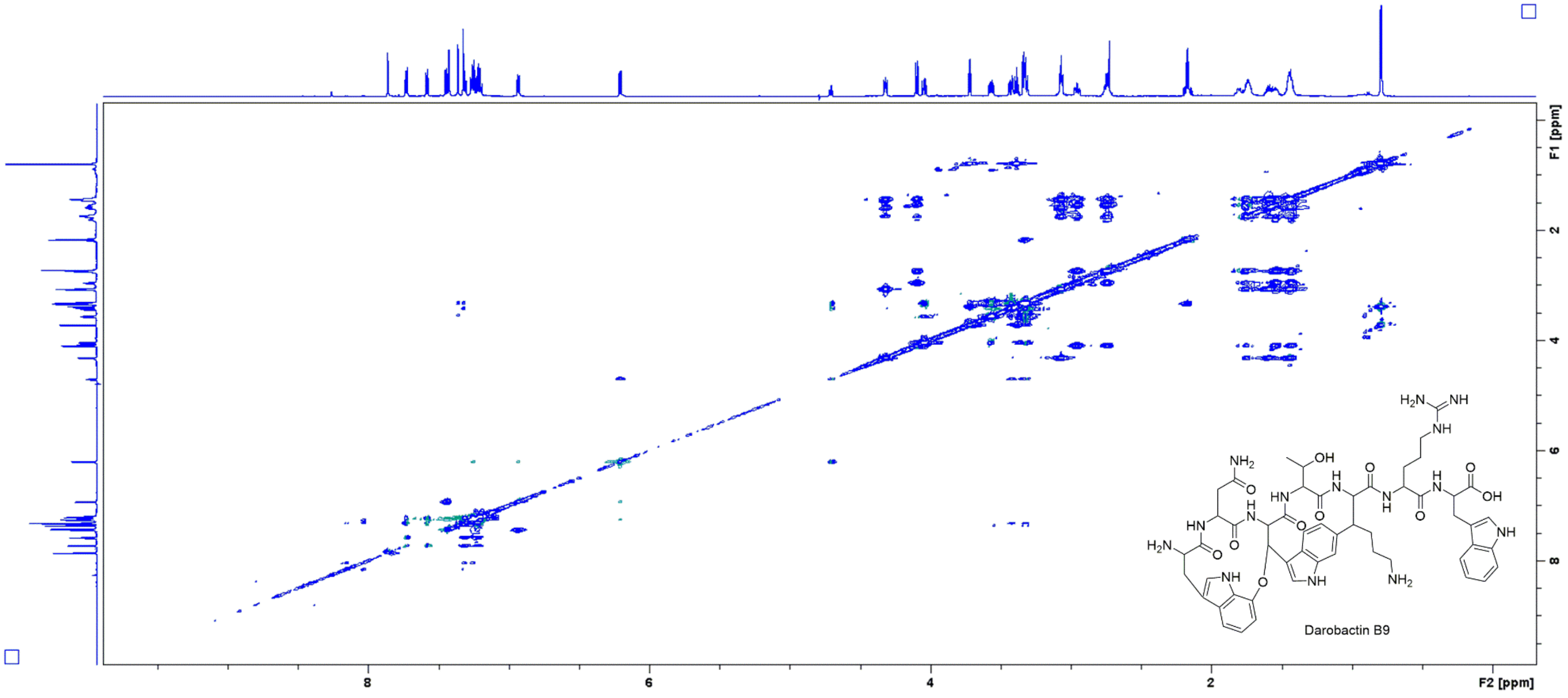

Figure S17: TOCSY spectrum of darobactin B9 (D_2O, 700 MHz), measured with H_2O suppression.

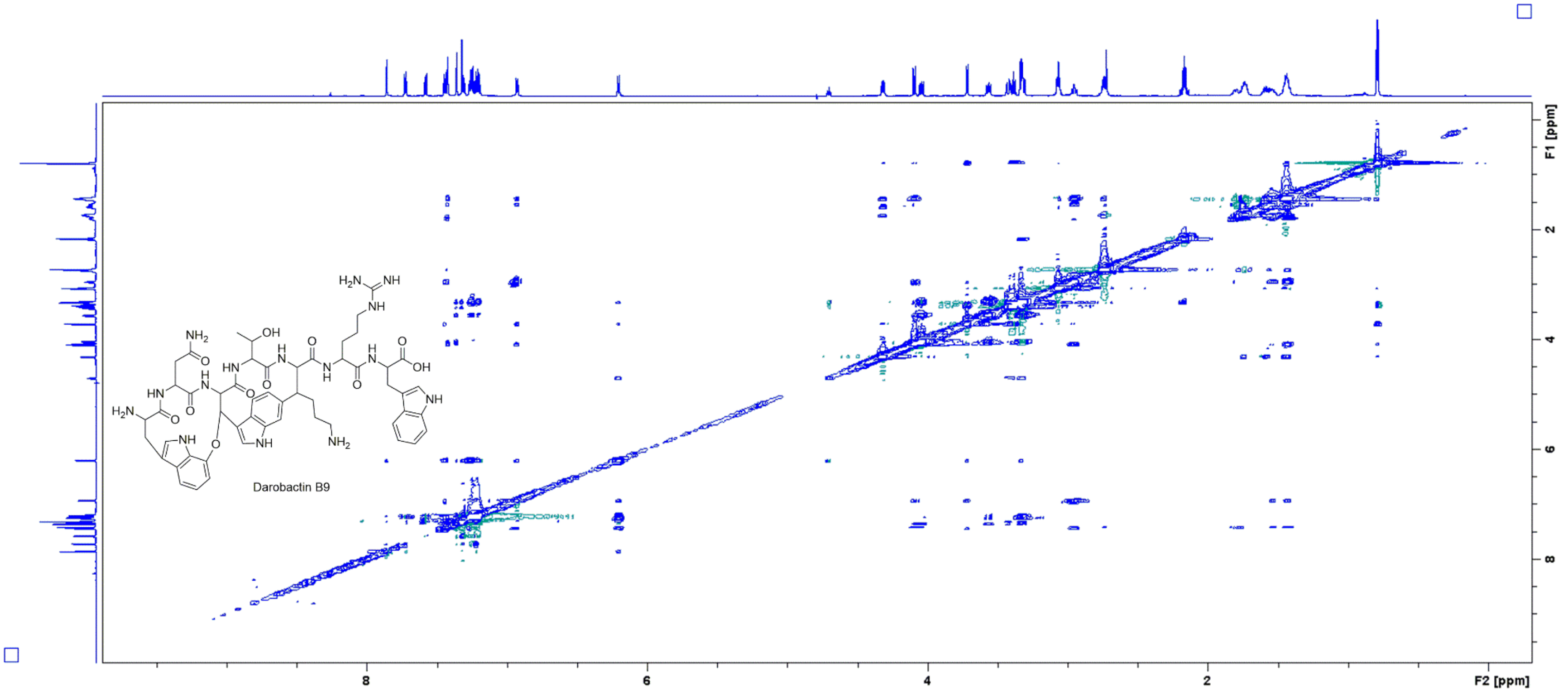

Figure S18: NOESY spectrum of darobactin B9 (D_2O, 700 MHz), measured with H_2O suppression.

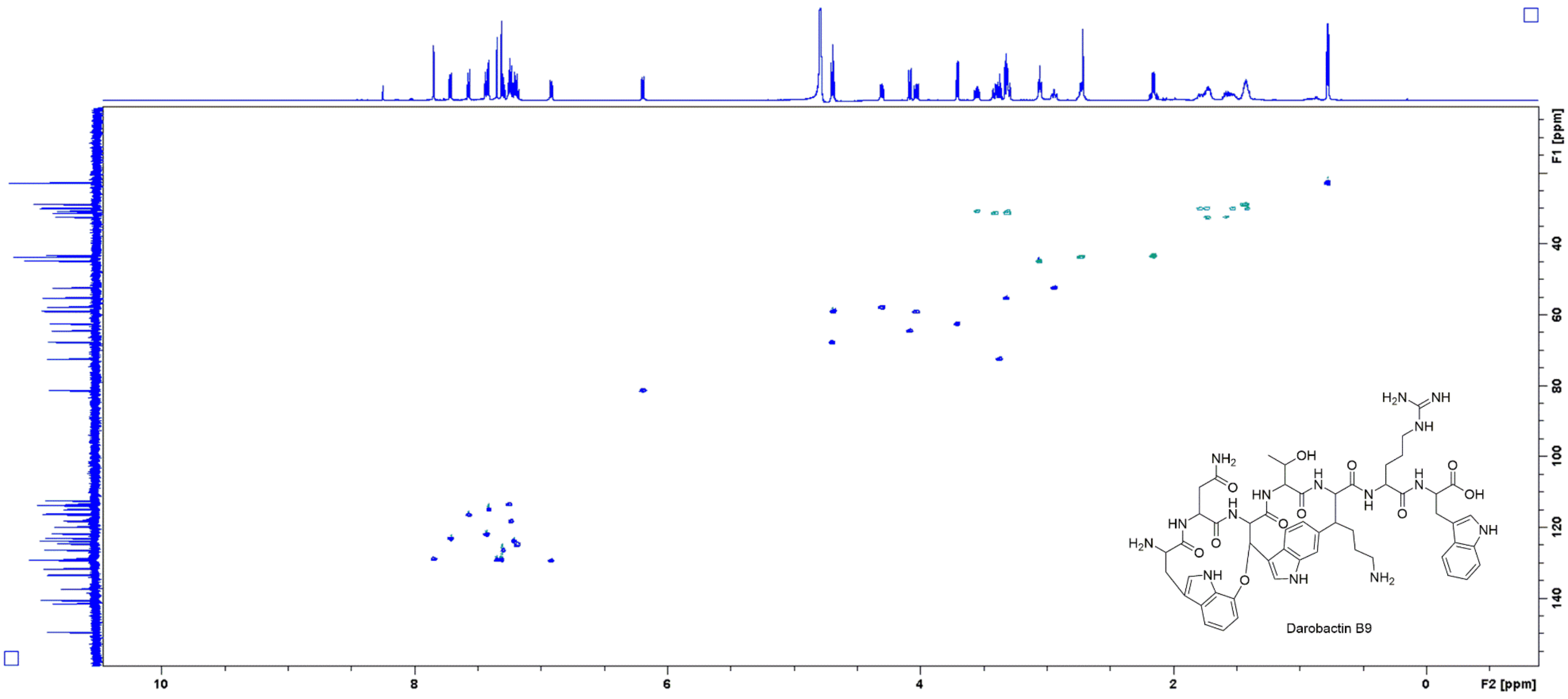

Figure S19: HSQC spectrum of darobactin B9 (D_2O, 600 MHz).

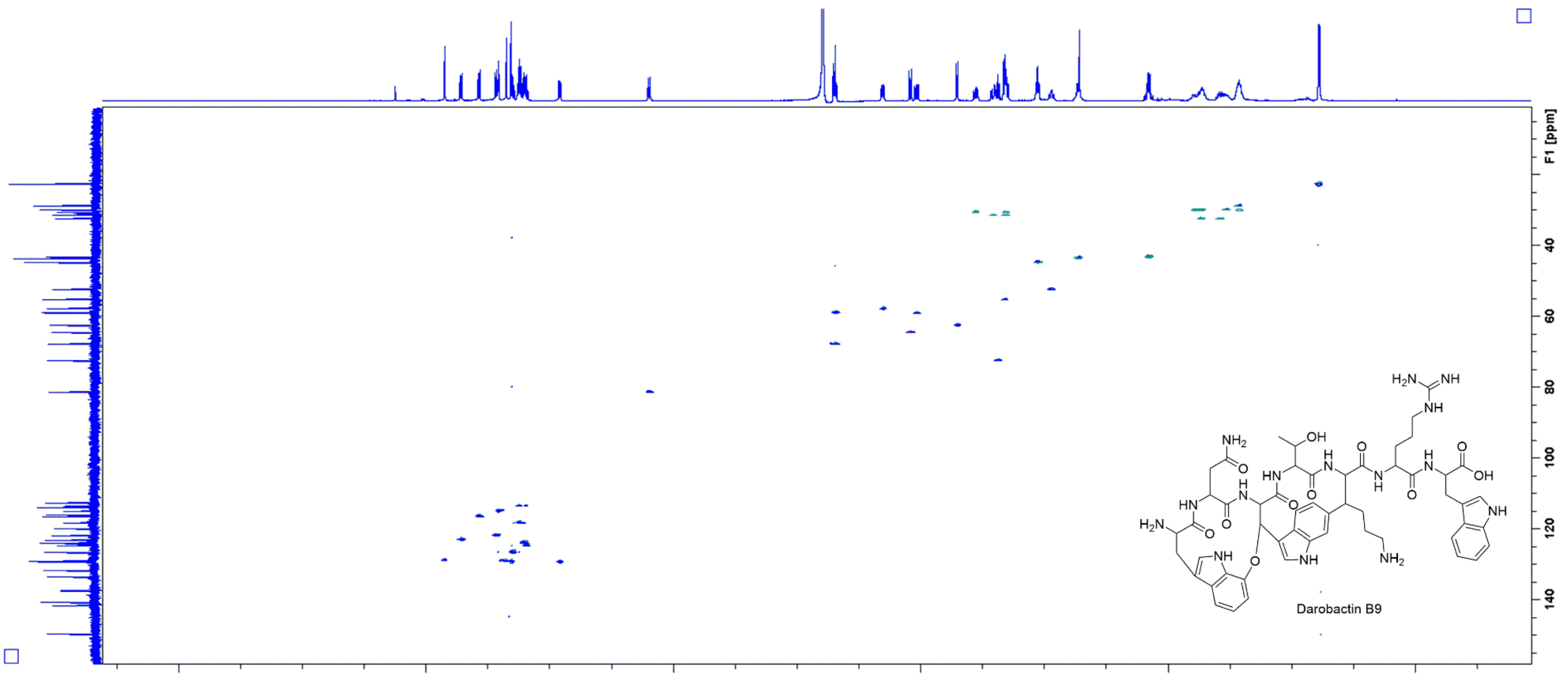

Figure S20: HSQC spectrum of darobactin B9 (D_2O, 600 MHz), measured with non-uniform sampling.

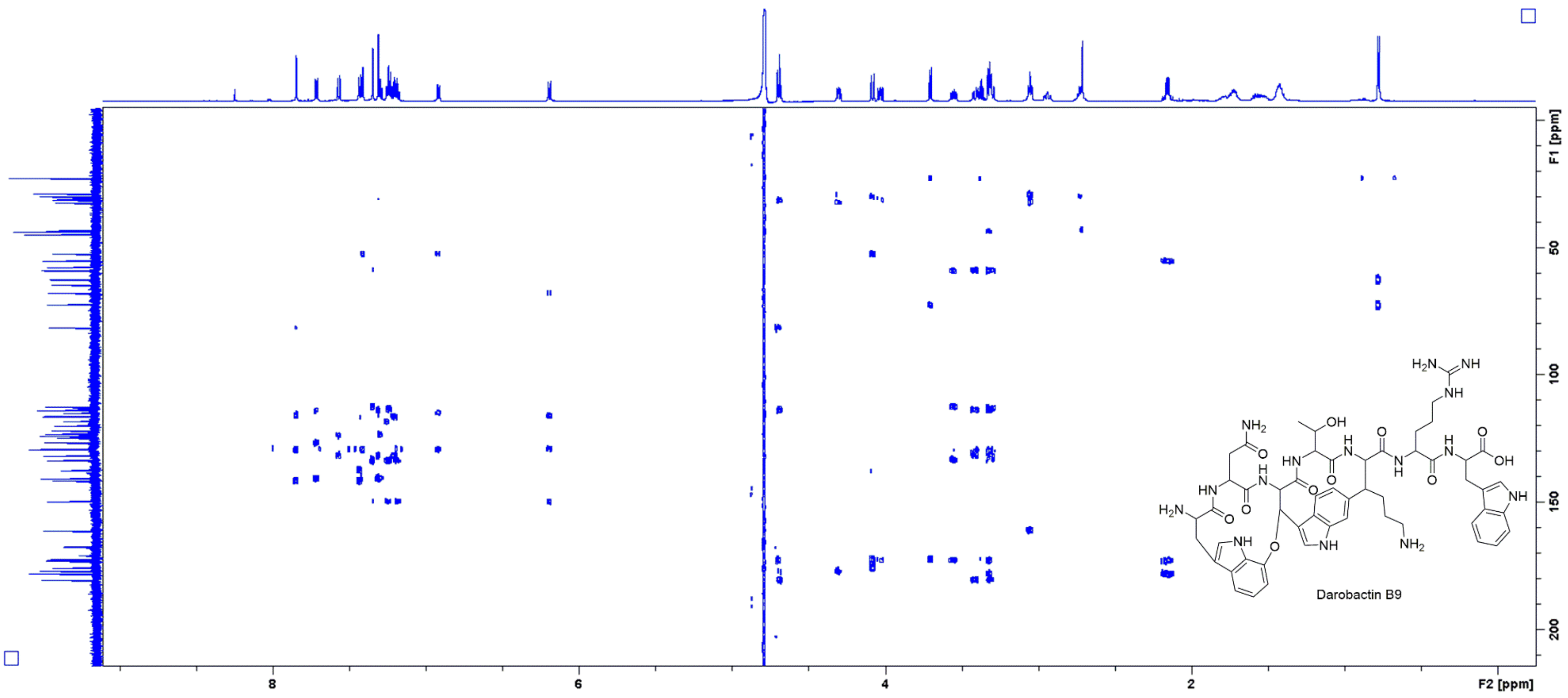

Figure S21: HMBC spectrum of darobactin B9 (D_2O, 600 MHz).

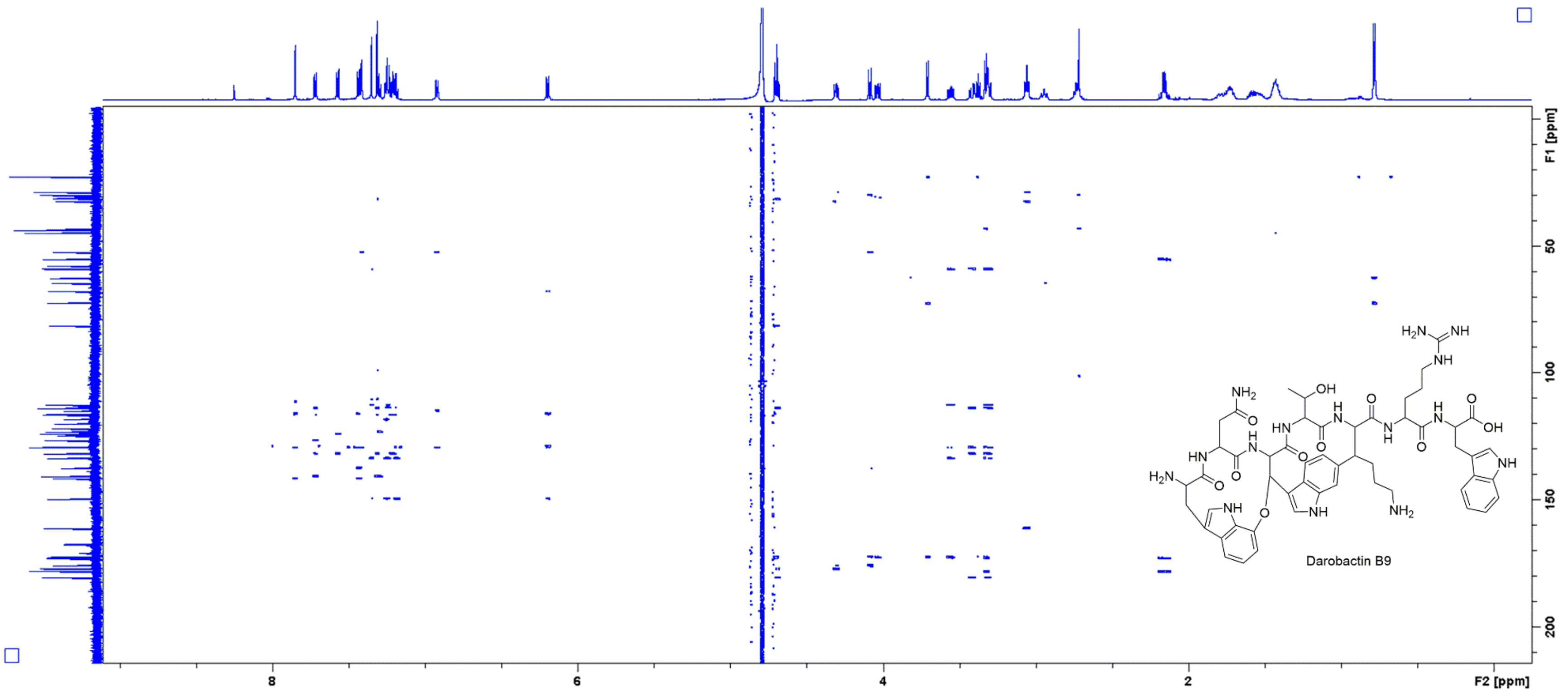

Figure S22: HMBC spectrum of darobactin B9 (D_2O, 600 MHz), measured with non-uniform sampling.
